# Establishment of gastrointestinal assembloids to study the interplay between epithelial crypts and their mesenchymal niche

**DOI:** 10.1038/s41467-023-38780-3

**Published:** 2023-05-25

**Authors:** Manqiang Lin, Kimberly Hartl, Julian Heuberger, Giulia Beccaceci, Hilmar Berger, Hao Li, Lichao Liu, Stefanie Müllerke, Thomas Conrad, Felix Heymann, Andrew Woehler, Frank Tacke, Nikolaus Rajewsky, Michael Sigal

**Affiliations:** 1 Department of Hepatology and Gastroenterology, Charité - Universitätsmedizin Berlin, 13353 Berlin, Germany; 2 Berlin Institute for Medical Systems Biology (BIMSB), Max Delbrück Center for Molecular Medicine in the Helmholtz Association (MDC), 10115 Berlin, Germany; 3 Genomics Technology Platform, Max Delbrück Center for Molecular Medicine in the Helmholtz Association (MDC), 13125 Berlin, Germany; 4 Core Facility Genomics, Berlin Institute of Health at Charité - Universitätsmedizin Berlin, 10178 Berlin, Germany

**Keywords:** Extracellular signalling molecules, Cell lineage, Intestinal stem cells

## Abstract

The cellular organization of gastrointestinal crypts is orchestrated by different cells of the stromal niche but available in vitro models fail to fully recapitulate the interplay between epithelium and stroma. Here, we establish a colon assembloid system comprising the epithelium and diverse stromal cell subtypes. These assembloids recapitulate the development of mature crypts resembling in vivo cellular diversity and organization, including maintenance of a stem/progenitor cell compartment in the base and their maturation into secretory/absorptive cell types. This process is supported by self-organizing stromal cells around the crypts that resemble in vivo organization, with cell types that support stem cell turnover adjacent to the stem cell compartment. Assembloids that lack BMP receptors either in epithelial or stromal cells fail to undergo proper crypt formation. Our data highlight the crucial role of bidirectional signaling between epithelium and stroma, with BMP as a central determinant of compartmentalization along the crypt axis.

## Abstract (web summary)

Most intestinal organoid models do not accurately model the interactions between epithelial and stromal cells. Here they establish a colon assembloid system with epithelial and stromal cells and demonstrate that BMP signals from differentiating epithelial cells promote mesenchymal niche organization.

## Introduction

The gastrointestinal epithelium is organized into clonal crypts that represent sophisticated anatomical and functional tissue units. The epithelial stem cells at the base of each colonic crypt constantly divide and self-renew, giving rise to short-lived differentiated lineages, such as absorptive colonocytes, secretory goblet cells, and enteroendocrine cells^1^. The epithelium is closely associated with a network of mesenchymal stroma, comprising fibroblasts, myofibroblasts, endothelial cells, pericytes, immune cells, and neural cells^2^. These diverse cell types have emerged as essential constituents of the stem cell niche that orchestrates epithelial stem cell homeostasis^2–8^. Using single-cell approaches, subsets of stromal fibroblasts have been identified as a critical source of growth factors and morphogens that promote epithelial stem cell self-renewal, proliferation, and directional cellular differentiation^2,9^.

Understanding the effects of niche-derived factors that control epithelial behavior has been made possible by organoid technology^10,11^. Gastrointestinal organoids can be derived from adult epithelial stem cells, with the stem cell niche mimicked by supplementing the culture with a cocktail of growth factors that activate selected signaling pathways like Wnt/β-catenin and epidermal growth factor (EGF)/mitogen-activated protein kinase (MAPK), while inhibiting others, such as bone morphogenetic protein (BMP)/Smad^10–13^. Exogenous supplementation with these growth factors is important for epithelial proliferation, stem cell self-renewal and robust long-term maintenance of organoid cultures. However, since the growth factors mainly target stem and progenitor cells, it is challenging to simultaneously obtain fully mature differentiated cell types, which have different niche requirements.

We hypothesized that crypt formation in vitro with simultaneous maintenance of proliferative stem cells as well as differentiated cells can be achieved by co-culturing epithelial cells with their stromal niche cells. Using this approach, we have developed a murine colon assembloid culture system that integrates the various primary cell types that build a functional colonic stem cell niche. Our assembloid system reveals that stromal cells are indeed capable of promoting the formation of colonic crypts that resemble the in vivo anatomy, cellular organization, signaling gradients, and directional epithelial turnover. We demonstrate that this requires bidirectional communication between the epithelium and its surrounding stroma: On the one hand, the stroma provides key growth factors required for crypt formation, while on the other hand, signals from the epithelium ensure diversification and anatomical positioning of stromal cell subsets—which together represent important prerequisites for mucosal self-organization.

## Results

### Colon assembloids recapitulate in vivo crypt organization

To investigate the crosstalk between epithelium and stromal cells in the context of crypt maturation and homeostasis, we developed an assembloid co-culture system that includes both components. Simply adding primary stromal cells to epithelial cells in a drop of Matrigel did not lead to a clear pattern of interaction between these two cell types or in vivo-like morphology, as stromal cells tended to migrate out of the Matrigel and adhere to the plate bottom (Supplementary Fig. 1a).

**Fig. 1:**
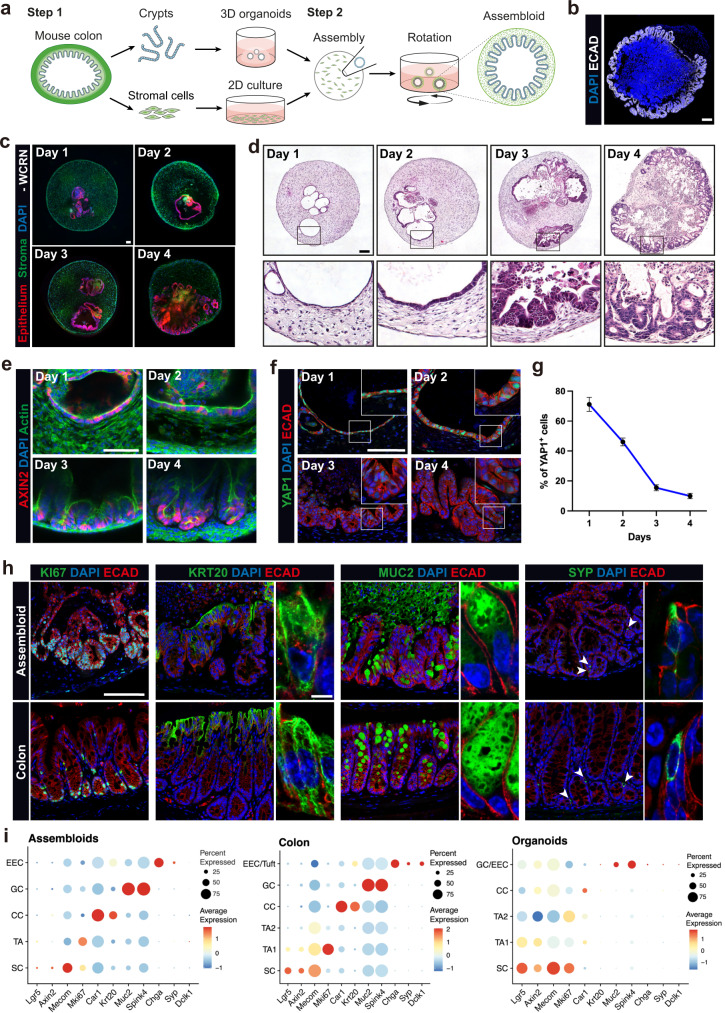
Establishment of colon assembloids modeling colonic crypt formation. **a** Schematic of colon assembloid generation (the schematic was created using Adobe Illustrator 26.3.1). **b** Immunofluorescence image of colon assembloids stained for E-cadherin (ECAD) and DAPI. Scale bar: 100 µm. **c** Confocal microscopy images of whole-mount staining of assembloids cultured in medium without sWnt, R-spondin 1, CHIR99021, or noggin (-WCRN) for 1–4 days. Epithelium was derived from *tg Act-DsRed* mice; stroma was derived from *tg Act-CFP* mice. Scale bar: 100 µm. **d** Hematoxylin and eosin (H&E) stained images of assembloids cultured in -WCRN medium for 1–4 days. Scale bar: 100 µm. **e** Confocal microscopy images of whole-mount staining for assembloids derived from *Axin2CreERT2/Rosa26-tdTomato* mice, cultured in -WCRN medium for 1–4 days, showing AXIN2 lineage tracing for 24 h. Scale bar: 100 µm. **f**, **g** Immunofluorescence images and quantification of colon assembloids cultured in -WCRN medium for 1–4 days stained for active YAP1 (*n* = 3 biological replicates per group). Scale bar: 100 µm. Data are presented as mean ± SEM. **h** Immunofluorescence images of colon assembloids and colon tissue labeled with markers for proliferative cells (KI67), colonocytes (KRT20), goblet cells (MUC2), and enteroendocrine cells (SYP). Scale bars: 100 µm (high magnification: 5 µm). **i** Dot plot showing expression of known marker genes against detected epithelial cell clusters identified by scRNA-seq in colon assembloids, tissue, and organoids. Circle size represents the within-cluster probability of gene detection. Fill color represents the normalized average expression level. EEC enteroendocrine cell, GC goblet cell, CC colonocyte, TA transit-amplifying cell, SC stem cell. scRNA-seq data are from two biological replicates per group. Images are representative of at least three biological replicates. Source data are provided as a Source Data file.

To overcome this, we developed a two-step process (Fig. 1a; Supplementary Fig. 1b, c). Using isolated murine colon crypts and stromal cells, we first generated three-dimensional (3D) epithelial organoids as well as primary stromal cell cultures as described previously^11,14^ (Fig. 1a). For assembly, we mixed the pre-cultured stromal cells into droplets of Matrigel and immediately added individual organoids into each droplet (Fig. 1a; Supplementary Figure 1b). To prevent migration of stromal cells to the bottom and ensure equal access of the entire assembloid surface to the medium, the cultures were grown on an orbital shaker (Fig. 1a; Supplementary Fig. 1b).

To monitor the epithelium and stroma over time in the culture, we assembled DsRed-labeled organoids with CFP-labeled stromal cells derived from *tg Act-DsRed* and *tg Act-CFP* mice, and cultured them in full 3D organoid medium (FM). Using live-cell fluorescent imaging or whole-mount staining with confocal microscopy, we were able to follow the spatiotemporal growth of the epithelial and stromal compartments (Supplementary Fig. 1d, e). Daily tracing of the assembloids demonstrated a rapid expansion of the epithelial compartment (Supplementary Fig. 1d, e). On day 4 of assembloid culture, the epithelium formed crypt-like structures surrounded by a tightly connected stromal layer (Supplementary Fig. 1f).

Since stromal cells provide important niche factors for intestinal crypts in vivo^4^, we asked whether epithelial crypt formation could be induced by factors produced by the stromal cells alone. Therefore, we removed Wnt activators (Wnt surrogate (W), CHIR-99021 (C), R-spondin 1 (R)), and the BMP inhibitor noggin (N) (WCRN) from the culture medium, normally required for stem cell maintenance in colon organoid cultures^11,15,16^. Despite the lack of such critical factors, we found that epithelial expansion in assembloids grown in WCRN-free (-WCRN) medium led to the formation of crypt-like structures that were comparable to those formed in full medium (FM) (Fig. 1b, c; Supplementary Fig. 1g). Since the supplementation of growth factors was dispensable, we employed the WCRN-free medium for subsequent experiments in the current study.

Next, we performed a time-course experiment to observe crypt formation in assembloids grown in -WCRN medium for 1 to 4 days (Fig. 1d). On day 1, the epithelium was structured as a thin monolayer (Fig. 1d). This was followed by epithelial cell thickening and organization of stromal cells that lined up parallel to the epithelium one day later (Fig. 1d). On day 3, we readily observed multiple epithelial invaginations with migration of stromal cells into the spaces between them, followed by the formation of true crypt-like structures on day 4 (Fig. 1d).

In vivo, colonic stem cells require high Wnt signaling activity, which is highest at the base of the crypt and induces the expression of the bona fide Wnt target gene AXIN2^3,17,18^. To visualize stem cells with active canonical Wnt signaling during crypt development, we used *Axin2CreErt2/Rosa26-tdTomato* lineage-tracing mice, as described previously^3,18^, generated assembloids and treated them with tamoxifen for 24 h to induce tdTomato expression in AXIN2-expressing cells in assembloids grown for 1 to 4 days (Fig. 1e). We found that tdTomato^+^ cells were spread over the epithelium on day 1 while the proportion of positive cell islets was reduced concurrent with epithelial expansion one day later (Fig. 1e). On day 3, tdTomato^+^ cells started to concentrate in the lower part of invaginations and were ultimately localized exclusively at the base of each crypt on day 4 (Fig. 1e). Our findings show a dynamic patterning of Wnt signaling activity during assembloid maturation and an in vivo-like AXIN2^+^ stem cell distribution in mature epithelia of assembloids cultured without supplementation of Wnt-inducing factors.

Yes-associated protein (YAP) is a transcriptional regulator that is largely inactive in the epithelium during homeostasis but is active during development and re-induced by injury, whereupon it drives a “fetal-like” regeneration process^19–22^. Using immunofluorescence labeling, we observed that in sections of adult mouse colon active YAP1 was indeed only present at low levels, whereas it was highly expressed in the nuclei of organoids grown in FM (Supplementary Fig. 1h), indicating that organoids resemble regenerative epithelium. Notably, assembloids also had high nuclear levels of active YAP1 at early stages during days 1 and 2, which was shut down on days 3 and 4, as crypts formed (Fig. 1f, g). These results demonstrate that assembloids mimic crypt maturation from “fetal-like” regenerative cells to normal adult crypts.

Next, we asked how different epithelial cell lineages organize during assembloid maturation. On day 1, epithelial cells were highly proliferative, as assessed by Ki67 staining, with only rare differentiated keratin 20 (KRT20)^+^ colonocytes, mucin 2 (MUC2)^+^ goblet cells, and synaptophysin (SYP)^+^ enteroendocrine cells (Supplementary Fig. 2a). By day 4, we observed a large number of differentiated cells, while Ki67^+^ cells were restricted to the crypt base. (Supplementary Fig. 2a). To assess whether the epithelial compartment from mature assembloids retains its stem cell potential, we passaged them and replated with fresh stromal cells. We found that epithelial cells readily expand and re-organize with stromal cells to form new assembloids with identical organization (Supplementary Fig. 2b).

To further explore how closely assembloids resemble the native tissue, we compared crypts in colon tissue with those in assembloids (Fig. 1h). We found a similar cellular organization, with cell proliferation restricted to the basal part of the crypts (Fig. 1h left), while surface colonocytes were located in the upper part (Fig. 1h second from left). In addition, assembloids contained numerous goblet cells and some enteroendocrine cells, resembling in vivo crypts (Fig. 1h). High-resolution imaging of colonocytes, goblet cells, and enteroendocrine cells in the assembloids revealed that they appeared fully differentiated and closely resembled the morphology of these cell types in vivo (see magnifications in Fig. 1h). Moreover, cleaved-caspase3-positive, apoptotic cells accumulated in the lumen (Supplementary Fig. 2c), indicating that cellular extrusion of short-lived cells into the lumen can also be reproduced in our model.

Notably, this degree of cellular self-organization could not be achieved in conventional colonic organoids: Most cells in organoids grown in FM were undifferentiated, similarly to epithelial cells in assembloids on day one (Supplementary Fig. 2a, d). While differentiation of organoids can easily be achieved by removing growth factors from the medium^16^, this approach enriches differentiated colonocytes at the expense of proliferation and stemness (Supplementary Fig. 2d), such that organoids represent a rather binary system—dominated by either stemness or differentiation.

To further characterize the epithelium in assembloids in comparison to organoids or in vivo colon tissue, we performed single-cell RNA sequencing (scRNA-seq), which enables the identification of all key cell types in the crypts. Epithelial cells in assembloids resembled the cellular diversity seen in the tissue to a high extent and showed a similar expression pattern as seen in vivo, confirming our morphological observations (Fig. 1i; Supplementary Fig. 3a–d). In contrast, colonic organoids—which require strong activation of Wnt signaling –were enriched for proliferative/stem cells and did not display the same degree of cellular diversity (Fig. 1i; Supplementary Fig. 3e, f). Together, these results demonstrate that colonic epithelium grown as assembloids faithfully mimics the epithelial cellular architecture and cell-type composition of the native tissue.

Similar to colon organoids, stomach organoids also require exogenous supplementation of Wnt ligands to maintain stem cell turnover. This Wnt treatment keeps stomach organoids in a highly proliferative state (KI67^+^) and allows them to differentiate into gland base secretory cells (GSII^+^), while MUC5AC^+^ pit mucous cells were rarely observed (Supplementary Fig. 4a). Moreover, our stomach organoids did not show an in vivo-like morphology but rather a cyst-like structure with only a few buds (Supplementary Fig. 4a). To explore whether the assembloid culture system could overcome this, we generated stomach assembloids and cultured them without Wnt (W), R-spondin (R), or noggin (N) (Supplementary Fig. 4b). Stomach assembloids formed gland-like structures after 6 days in WRN-free medium (Supplementary Fig. 4b). We then visualized gastric epithelial cell types in stomach assembloids and stomach tissue by immunofluorescence (Supplementary Fig. 4c). As with colon assembloids, stomach assembloids showed an in vivo-like cell organization, with GSII^+^ secretory cells at the gland base and KI67^+^ proliferative cells at the isthmus, while MUC5AC^+^ pit cells were located in the upper gland (Supplementary Fig. 4c).

### Stromal cellular diversity in colon assembloids

**Fig. 2:**
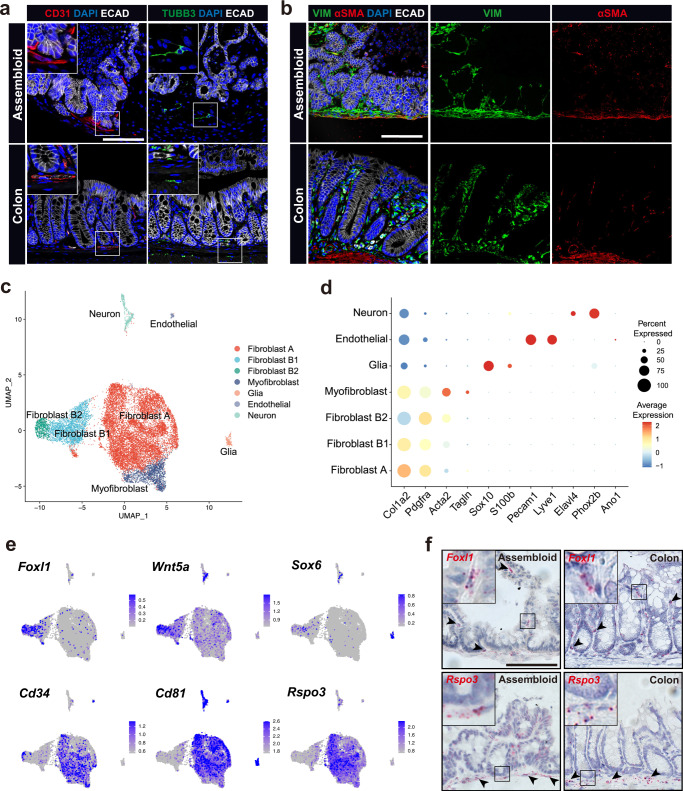
Stromal cellular complexity of colon assembloids. **a** Immunofluorescence images of day 4 colon assembloids and tissue stained for endothelial cells (CD31) and neuronal cells (TUBB3). **b** Immunofluorescence images of day 4 colon assembloids and tissue stained for fibroblast (VIM) and myofibroblast (αSMA) markers. **c** UMAP plot of scRNA-seq dataset from colon assembloid stromal cells. **d** Dot plot showing expression of known marker genes against detected stromal clusters in colon assembloids identified by scRNA-seq. Circle size represents the within-cluster probability of gene detection. Fill color represents the normalized average expression level. **e** UMAP expression plots of telocyte marker genes (*Foxl1*, *Wnt5a*, and *Sox6*) and trophocyte marker genes (*Cd34*, *Cd81*, and *Rspo3*) in the assembloid dataset. Cells colored by normalized expression of indicated marker genes. **f** Single-molecule in situ hybridization (sm-ISH) showing localization of *Foxl1* and *Rspo3* in assembloids and colon tissue. Scale bars: 100 µm. scRNA-seq data are from two biological replicates per group. Images are representative of at least three biological replicates.

Our data indicated that stromal cells support epithelial crypt formation and cellular organization. To shed light on the cellular diversity and organization of the stroma of colon assembloids, we visualized several key stromal cell types using immunofluorescence and compared them with those in colon tissue. On day 4, assembloids had maintained CD31^+^ endothelial cells, which were organized similarly to those in native tissue (Fig. 2a; Supplementary Fig. 5a). β3-tubulin (TUBB3) staining revealed the presence of neuronal cell bodies without processes on day one, while on day 4, they had extended axonal processes into the stromal compartment similarly to the pattern observed in the colon (Fig. 2a; Supplementary Fig. 5a). Vimentin (VIM)^+^ fibroblasts and α-SMA^+^ myofibroblasts showed a disordered distribution on day 1 (Supplementary Fig. 5a), while on day 4 VIM^+^ fibroblasts were located beneath and between crypts in close contact with epithelium, whereas α-SMA^+^ myofibroblasts were found mainly in the outer layer, similar to the lamina muscularis mucosae in the colon (Fig. 2b; Supplementary Fig. 5a).

Next, we performed scRNA-seq for non-epithelial (EpCAM-negative) cells from assembloids. Graph-based clustering revealed seven cell clusters (Fig. 2c, d; Supplementary Fig. 5b): Fibroblast populations Fibroblast A and B1/B2 (*Col1a2*^+^ *Pdgfra*^+^), myofibroblasts (*Acta2*^+^ *Tagln*^+^), glial cells (*Sox10*^+^ *S100b*^+^), endothelial cells (*Pecam1*^+^ *Lyve1*^+^), and neurons (*Elavl4*^+^ *Phox2b*^+^). To compare the stromal cell types in assembloids and colon tissue, we analyzed integrated data from two published in vivo datasets^2,23^, which confirmed the presence of the key subsets of cells found in vivo (Supplementary Fig. 6a, b). Previous studies have identified subsets of fibroblasts in the small intestine with distinct functional features^9^. Based on their expression pattern, these cells were termed telocytes, expressing high levels of *Pdgfra* and forkhead box L1 (*Foxl1*) and trophocytes, expressing *Cd34* and *Cd81*. In the colon, we identified three subsets of cells, with Fibroblast 2 resembling telocytes, and Fibroblast 1 and 3 resembling features of trophocytes (Supplementary Fig. 6a–d).

In our assembloids, based on expression of the marker genes *Cd34*, *Cd81*, and *Foxl1*^2,9,14,24^ we identified the Fibroblast A cluster as trophocyte-like cells (*Cd34*^+^ *Cd81*^+^ *Foxl1*^-^) and Fibroblast B1/2 as telocyte-like cells (*Cd34*^-^ *Cd81*^-^ *Foxl1*^+^) (Fig. 2e). Fibroblast A was also marked by the Wnt agonist R-spondin 3 (*Rspo3*) (Fig. 2e; Supplementary Fig. 5c), a key driver of stem cell self-renewal^3,25^, while Fibroblast B1/2 expressed the non-canonical Wnt ligand *Wnt5a* and *Sox6* (Fig. 2e; Supplementary Fig. 5c), which is in line with in vivo findings (Supplementary Fig. 6c, d)^2,9^. Of note, Gene Set Variation Analysis showed some heterogeneity within the Fibroblast B clusters, with Fibroblast B2 in assembloids most closely resembling Fibroblast 2 in the colon, while Fibroblast B1 in assembloids shows some features of Fibroblast 1 as well as Fibroblast 2 in the colon (Supplementary Fig. 6e).

We next assessed the spatial distribution of the fibroblast populations in assembloids using single-molecule in situ hybridization (sm-ISH) for *Foxl1* and *Rspo3* (Fig. 2f). *Foxl1* expression (Fibroblast B1/B2 in assembloids, Fibroblast 2 in the colon) was located primarily along the crypt axis with only a few cells in close proximity to the epithelium at the crypt base. *Rspo3* expression (Fibroblast A in assembloids, Fibroblast 1/3 in the colon) was concentrated beneath the crypts. Overall, these data demonstrate that assembloids closely mimic not only the epithelial but also the mesenchymal architecture of colonic crypts in vivo, recapitulating the diversity and spatial distribution of stromal cells including telocyte- and trophocyte-like cells.

### Assembloids resemble BMP molecule expression in the colon

**Fig. 3:**
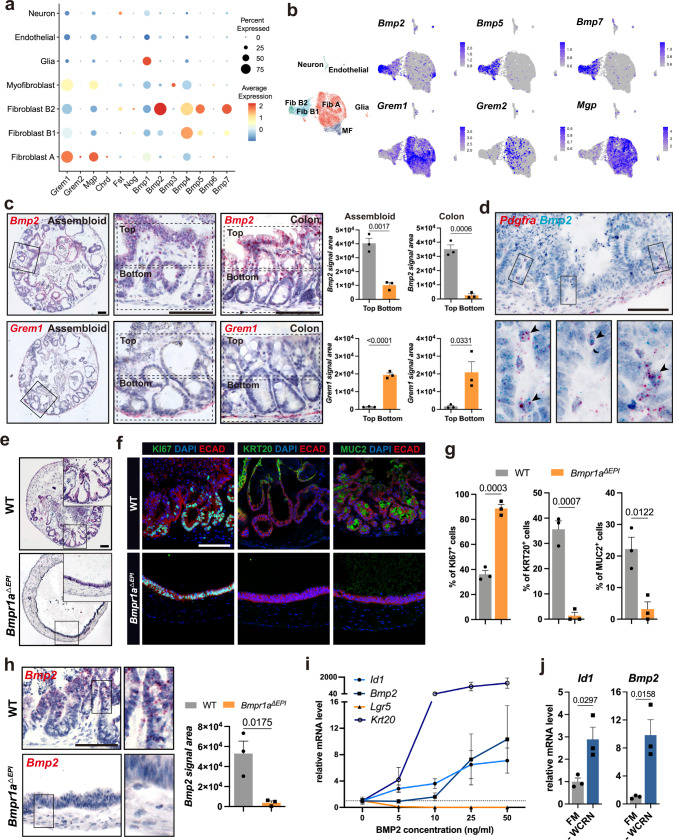
BMP gradient in colon assembloids and its effect on colonic crypt formation. **a** Dot plot showing expression of BMP signaling molecules in the identified stromal cell clusters from assembloids. **b** UMAP expression plots of BMP ligand genes (*Bmp2*, *Bmp5*, and *Bmp7*) and BMP antagonist genes (*Grem1*, *Grem2*, and *Mgp*) for stromal cells in assembloids. **c** Single-molecule in situ hybridization (sm-ISH) using serial sections showing localization of *Bmp2* and *Grem1* in assembloids and colon tissue; quantification for *Bmp2* and *Grem1* signal area divided into top and bottom in the crypt (*n* = 3 biological replicates per group). **d** Duplex sm-ISH showing co-localization of *Pdgfra* and *Bmp2* in assembloids. **e** Hematoxylin and eosin (H&E) staining of an assembloid derived from a wild-type mouse (WT) and an assembloid comprised of epithelial cells from an *Axin2CreErt2/Bmpr1a*^*fl/fl*^ mouse and WT mesenchymal cells from a WT mouse (*Bmpr1a*^*ΔEPI*^). **f**, **g**) Immunofluorescence images and quantification of WT and *Bmpr1a*^*ΔEPI*^ assembloids stained for KI67, KRT20, and MUC2 (*n* = 3 biological replicates per group). **h** sm-ISH images and quantification of *Bmp2* expression in WT and *Bmpr1a*^*ΔEPI*^ assembloids (*n* = 3 biological replicates per group). **i** qPCR for expression of the stem cell marker *Lgr5*, the colonocyte marker *Krt20*, the BMP ligand *Bmp2*, and the BMP target *Id1* from organoids cultured with different concentrations of BMP2 (*n* = 3 biological replicates per group). **j** qPCR for expression of *Id1* and *Bmp2* from organoids cultured in full medium (FM) or in medium without sWnt, R-spondin 1, CHIR99021, or noggin (-WCRN) (*n* = 3 biological replicates per group). Scale bars: 100 µm. Data are presented as mean ± SEM. Statistical analyses were performed using Student’s *t*-test (two-tailed) for (**c**, **g**, **h**), and (**j**). Fib Fibroblast, MF Myofibroblast. scRNA-seq data are from two biological replicates per group. Images are representative of at least three biological replicates. Source data are provided as a Source Data file.

Telocytes and trophocytes express molecules with opposing effects on BMP signaling^9^. To explore the functionality of the identified telocyte- and trophocyte-like cells in our assembloids, we analyzed the expression of factors that regulate BMP signaling. Consistent with in vivo data (Supplementary Fig. 6f), BMP antagonists including gremlin 1 (*Grem1*), gremlin 2 (*Grem2*), and matrix Gla protein (*Mgp*) were indeed expressed in Fibroblast A, and BMP agonists such as *Bmp2*, *Bmp5*, and *Bmp7*, were almost exclusively expressed in Fibroblast B1/B2 (Fig. 3a, b). *Bmp2* and *Grem1* are highly expressed in the colon tissue and show a distribution that is in line with the well-explored gradient of BMP signaling towards the top of the crypt^2,26,27^.

We thus explored the local expression of these two molecules using sm-ISH on serial sections of assembloids and colon tissue. In both, *Bmp2* was expressed predominantly at the top of crypts in epithelial as well as stromal cells, gradually decreasing towards the base (Fig. 3c). Both assembloids and colon also showed a similar expression pattern of the BMP inhibitor *Grem1*, a specific trophocyte marker^9^, which was expressed in the subcryptal stromal cell compartment (Fig. 3c). Duplex ISH for *Bmp2* and *Pdgfra* in assembloids confirmed that in addition to surface epithelial cells, *Pdgfra*^+^ fibroblasts located in the intercrypt regions indeed expressed *Bmp2*. Furthermore, we visualized the BMP signaling target gene *Id1* in assembloids and colon tissue, both of which showed enriched expression at the upper crypt but not at the base (Supplementary Fig. 7a).

### Epithelial BMP promotes differentiation and crypt formation

BMP is a key factor that drives cell differentiation in the gastrointestinal tract^28^. To explore the functionality of our model for investigating the signaling between epithelium and stroma in detail, we analyzed the role of BMP signaling in establishing crypt cell organization. BMP receptor type 1A (BMPR1A), with a high affinity for BMP2, has been widely utilized to study the role of BMP signaling in the gastrointestinal tract^28^. We thus generated assembloids from wild-type (WT) stromal cells and *Bmpr1a* knock-out (KO) epithelial cells (*Bmpr1a*^*ΔEPI*^) that were derived from *Axin2*^*CreErt2*^*/Bmpr1a*^*fl/fl*^ mice. These assembloids failed to compartmentalize and remained as simple spheroids lined with an epithelial monolayer (Fig. 3e). In addition to the defective crypt formation, epithelial cells also failed to differentiate, as assessed by expression of KRT20 and MUC2 (Fig. 3f, g). In contrast, the pan-proliferative marker KI67 was highly expressed throughout the epithelium (Fig. 3f, g). Thus, we confirmed in assembloids the critical role of BMP signaling for epithelial differentiation.

*Bmpr1a*^*ΔEPI*^ assembloids showed significantly lower levels of *Bmp2* expression than WT assembloids (Fig. 3h). Additionally, *Bmp2* was expressed in differentiated surface cells in the colon in vivo as well as in the WT assembloids (Fig. 3c, h). Indeed using organoids we found that *Bmp2* expression was low in organoids grown in FM but was induced when organoids were differentiated by addition of BMP2 or culture in WCRN-free medium (Fig. 3i, j; Supplementary Fig. 7b–d), which is consistent with our recent findings in the stomach showing a positive feed-forward loop of BMP signaling in epithelial cells^29^.

### BMP shapes the identity and function of the stromal niche

The spatial distribution of BMP signaling molecules led us to ask whether and how BMP may affect the mesenchymal compartment during assembloid growth. Sm-ISH showed that in early cultures, the expression level of *Bmp2* was low, and *Foxl1*^+^ *Wnt5a*^+^ telocyte-like cells were absent, while *Grem1* was broadly expressed in the stromal compartment (Supplementary Fig. 7e). As mature crypts formed, *Bmp2* production was augmented at the top of the crypts, while stromal cells in the inter-crypt region expressed *Foxl1* and *Wnt5a* but no *Grem1* (Supplementary Fig. 7e), suggesting a potential relationship between BMP signaling and stromal cell organization.

**Fig. 4:**
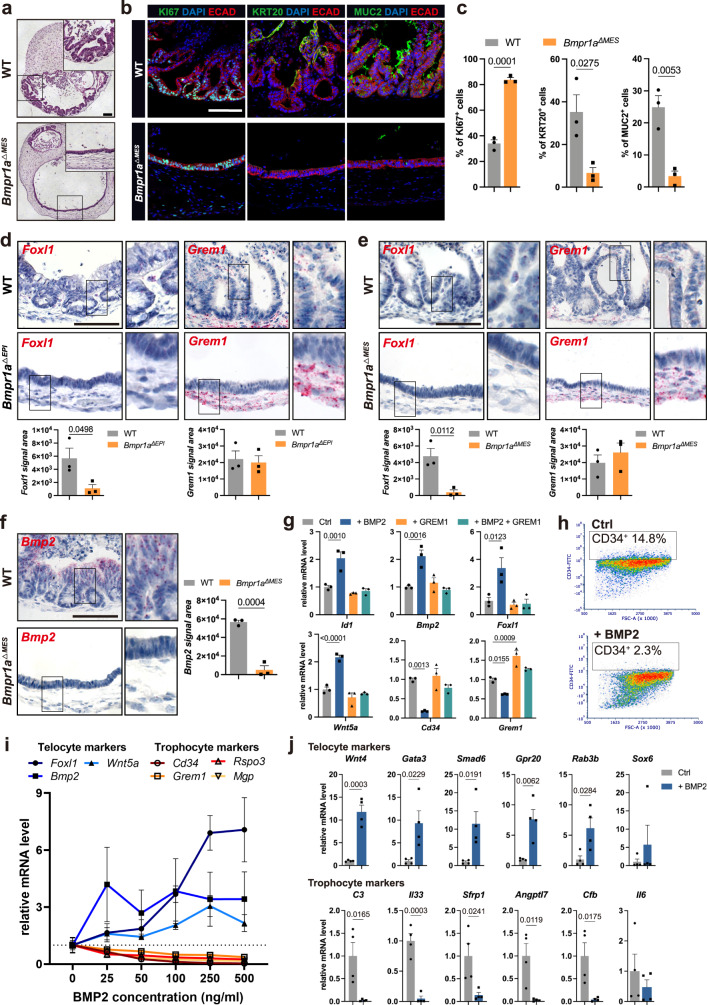
BMP signaling promotes functional transition of stromal cells. **a** Hematoxylin and eosin (H&E) staining of a WT assembloid and an assembloid comprising *Bmpr1a* KO mesenchymal cells derived from a *Bmpr1a*^*fl/fl*^ mouse and WT epithelial cells from a WT mouse (*Bmpr1a*^*ΔMES*^). **b**, **c** Immunofluorescence images and quantification of WT and *Bmpr1a*^*ΔMES*^ assembloids stained for KI67, KRT20, and MUC2 (*n* = 3 biological replicates per group). **d** sm-ISH images and quantification of *Foxl1* and *Grem1* expression in WT and *Bmpr1a*^*ΔEPI*^ assembloids (*n* = 3 biological replicates per group). **e** sm-ISH images and quantification of *Foxl1* and *Grem1* expression in WT and *Bmpr1a*^*ΔMES*^ assembloids (*n* = 3 biological replicates per group). **f** sm-ISH images and quantification of *Bmp2* expression in WT and *Bmpr1a*^*ΔMES*^ assembloids (*n* = 3 biological replicates per group). **g** qPCR for expression of *Id1*, telocyte marker genes (*Bmp2*, *Foxl1*, and *Wnt5a*), and trophocyte marker genes (*Cd34* and *Grem1*) of primary murine colon stromal cells cultured in normal medium, or treated with 50 ng/ml BMP2, 500 ng/ml GREM1, or 50 ng/ml BMP2 and 500 ng/ml GREM1 (*n* = 3 biological replicates per group). **h** Flow cytometry analysis of EpCAM^-^ CD45^-^ CD31^-^ CD34^+^ cell fractions in primary murine colon stromal cells cultured in normal medium or treated with 50 ng/ml BMP2. **i** qPCR for expression of telocyte and trophocyte marker genes in EpCAM^−^ CD45^−^ CD31^−^ CD34^+^ primary murine colon stromal cells exposed to the indicated BMP2 concentrations (*n* = 3 biological replicates per group). **j** qPCR for expression of additional telocyte and trophocyte marker genes of EpCAM^−^ CD45^−^ CD31^−^ CD34^+^ primary murine colon stromal cells exposed to 250 ng/ml BMP2 (*n* = 4 biological replicates per group). Scale bars: 100 µm. Data are presented as mean ± SEM. Statistical analyses were performed using Student’s *t* test (two-tailed) for (**c**–**f**) and (**j**); using one-way ANOVA, followed by Tukey’s multiple comparisons test for (**g**). Images are representative of at least three biological replicates. Source data are provided as a Source Data file.

To explore this, we generated assembloids from *Bmpr1a* KO mesenchymal stromal cells and WT epithelial cells (*Bmpr1a*^*ΔMES*^). Similar to the *Bmpr1a*^*ΔEPI*^ assembloids, the epithelial cells failed to form proper crypt structures and differentiate (Fig. 4a–c), suggesting that crypt maturation requires activation of BMP signaling in the stroma. Visualizing trophocyte- and telocyte-like cells in *Bmpr1a*^*ΔEPI*^ and *Bmpr1a*^*ΔMES*^ assembloids by sm-ISH (Fig. 4d, e) revealed that both *Bmpr1a*^*ΔEPI*^ and *Bmpr1a*^*ΔMES*^ assembloids expressed only low level of the telocyte-specific gene *Foxl1* (Fig. 4d, e). Moreover, we again observed greatly diminished *Bmp2* expression in both epithelium and stroma of *Bmpr1a*^*ΔMES*^ assembloids, as found in *Bmpr1a*^*ΔEPI*^ assembloids (Fig. 4f).

To study the impact of BMP2 on mesenchymal stromal cells, we treated primary colon stromal cells with BMP2. qPCR confirmed their responsiveness by upregulated expression of *Id1* (Fig. 4g). We noticed that similar to the epithelium, BMP2 also triggered the expression of *Bmp2* (Fig. 4g). Remarkably, we found that the telocyte markers *Foxl1* and *Wnt5a* were both significantly increased, while the trophocyte markers *Cd34* and *Grem1* were downregulated after BMP2 treatment (Fig. 4g). These effects could be reversed by adding the BMP inhibitor GREM1 to the culture (Fig. 4g). Accordingly, flow cytometry revealed a reduction in the proportion of EpCAM^-^ CD45^-^ CD31^-^ (non-epithelial, non-immune, non-endothelial) CD34^+^ trophocyte-like cells upon BMP2 treatment (Supplementary Fig. 7f; Fig. 4h).

To further dissect the effect of BMP2 on trophocyte-like cells, we performed fluorescence-activated single-cell sorting (FACS) to isolate EpCAM^-^ CD45^-^ CD31^-^ CD34^+^ stromal cells from mouse colon and exposed them to BMP2 (Supplementary Fig. 7g; Fig. 4i, j). qPCR showed that BMP2 led to a significant decrease in the expression of trophocyte (Fibroblast 1/3) marker genes, while the expression of telocyte (Fibroblast 2) marker genes was increased (Supplementary Fig. 7h; Fig. 4i, j). Together, these results demonstrate stromal responses to BMP signaling and indicate that the spatial distribution of stromal cell subsets is determined by local signals from epithelium.

### Generation of colon tumor assembloids

**Fig. 5:**
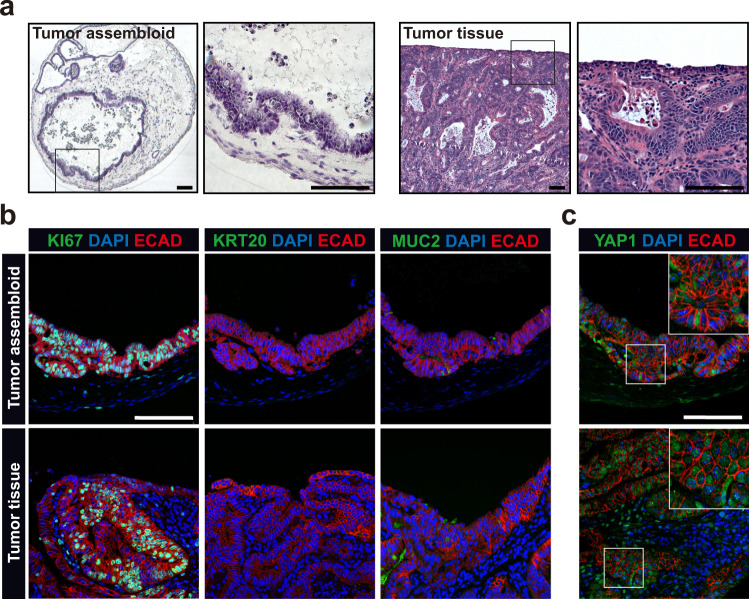
Establishment of colon tumor assembloids. **a** Hematoxylin and eosin (H&E) staining of a colon tumor assembloid and colon tumor tissue derived from the AOM/DSS mouse model. **b** Immunofluorescence images of a tumor assembloid and tumor tissue stained for KI67, KRT20, and MUC2. **c** Immunofluorescence images of a tumor assembloid and tumor tissue stained for active YAP1. Scale bar: 100 µm. Images are representative of at least three biological replicates.

To investigate whether assembloids can also be used to model intestinal tumorigenesis, we induced colitis-associated tumors in mice using the previously established azoxymethane/dextran sodium sulfate (AOM/DSS) model (Supplementary Fig. 8a, b)^30,31^. We first grew organoids from the tumor tissue and found that they exhibited Wnt-, CHIR-99021-, and noggin-independent growth (Supplementary Fig. 8c, d). We then generated tumor assembloids using tumor organoids and WT stromal cells. Colon tumor cells in assembloids grew as irregular ridges rather than typical crypt-like structures, similar to the tumor morphology in vivo (Fig. 5a), which we did not see when we cultured tumors as organoids (Supplementary Fig. 8d). As with tumor cells in vivo, most epithelial cells in the assembloids were highly proliferative, and differentiation was impaired (Fig. 5b). We noticed high levels of active nuclear YAP1 in both tumor assembloids and tissue (Fig. 5c), indicative of a more primitive state with fetal-like properties^32^. Overall, these results show that the assembloid model is also able to capture the morphological characteristics of colon tumors and could be used to study the interaction between tumors and their microenvironment.

## Discussion

Here we have developed an assembloid culture system that enables the study of intercellular communication and self-organization of gastrointestinal tissues. The system allows dissection of the molecular signals provided by stromal cells along the crypt axis that control epithelial proliferation and differentiation, as well as vice versa the influence of epithelial signals on stromal cells. By recapitulating the processes that coordinate epithelial self-organization and maturation, the assembloid system enables the investigation of gastrointestinal mucosal development and homeostasis in vitro and will be able to rapidly drive a better understanding of processes responsible for tissue dysfunction and malignant transformation.

Currently, gastrointestinal organoids represent the gold standard for studying epithelial stem cell behavior in the gastrointestinal tract^33^. The organoid system has revolutionized biomedical research and also lends itself to the modeling of gastrointestinal disorders^34,35^. While organoids mimic intestinal epithelial self-organization to a certain degree^10^, they mostly resemble the crypt base stem cell compartment^11^. Although differentiation can be induced by withdrawal of growth factors, the differentiation capacity of organoids is limited, and the stem cell compartment is lost in differentiation conditions, thus preventing the modeling of the full spectrum of crypt cell states. This is particularly true for colon and stomach organoids, which, in contrast to organoids from the small intestine, rely on high Wnt supplementation, resulting in a highly proliferative, immature epithelium^11,29^. Our assembloids enable the generation of a culture that resembles mature crypts and contains both adult stem cells as well as differentiated cells, which results from the reciprocal interplay between the stroma and the epithelium in the establishment of gastrointestinal crypts. Assembloids thus anatomically resemble the in vivo state more closely and comprehensively than conventional organoids and allow investigation of the processes that are responsible for the formation and maintenance of the signaling gradients guiding cellular differentiation along the crypt axis.

The high proliferative activity of the gastrointestinal epithelium makes it challenging to establish an ideal culture condition that includes less proliferative cells from the stroma. Recently, several groups have used co-culture systems with defined gastrointestinal cell subsets such as epithelial cells and fibroblasts/myofibroblasts^36,37^, vessels^38^, immune cells^39^, or neural cells^40,41^ derived from adult primary cells or pluripotent-stem-cells. These systems used either air-liquid interface (ALI) cultures with stromal cells separated from the epithelium or relied on the traditional organoid technique, with the additional cell types added to the static Matrigel drop. Both approaches have provided important insights but fail to fully recapitulate the interplay between epithelium and stroma or the emergence of mature crypts in vitro. Our system allows the expansion of epithelial cells in direct contact with stromal cells and we reveal that this close interplay is a crucial basis for crypt maturation and the corresponding organization of the stroma.

A previous study has reported the generation of assembloids from the urinary bladder as well as corresponding tumors^42^. These multilayer assembloids also exhibited characteristics of the mature adult tissue and elucidated the determination of tumor plasticity induced by stromal cells. However, unlike our colon assembloids, the stromal compartment of the bladder assembloid system was derived from heterologous origins, including mouse embryonic fibroblasts and the human lung microvascular endothelial cell line-5a (HULEC-5a). These cell lines indeed also supported the formation of organotypic structures by the epithelium. In our assembloid system, we used primary stromal cells from homologous tissue and reveal remarkable anatomical self-organization of these cells as well the underlying signals. Notably, primary stromal cells in our culture provide the Wnt activators and BMP inhibitors necessary for maintaining colon stem cells, making the colon assembloids self-sufficient with regard to these essential signaling pathways.

Subsets of fibroblasts in the intestine play a critical role in regulating epithelial cell proliferation and differentiation^2,9,14,23^. The two main subsets of mesenchymal fibroblasts in the small intestine can be separated by their expression pattern of either BMP inhibitors in trophocytes or BMP ligands in telocytes^9^. Although several studies have named the cells differently^2,23^, the functional separation of BMP-ligand-producing and BMP-inhibitor-producing cells can be recapitulated in the small intestine and colon. In the current study, we re-identify these two fibroblast populations in colon assembloids. Our data suggest that the mesenchymal cells respond to BMP signals from the differentiating epithelium and that activation or suppression of BMP signaling in the stroma itself may determine the cellular distribution of niche cell subsets. Interestingly, recent publications from the Shivdasani lab indicate that BMPs derived from telocytes (also called subepithelial myofibroblasts) not only drive epithelial differentiation^43^ but also repress stem cell niche factors such as *Grem1* and *Rspo3* in neighboring mesenchymal cells^44^. Our findings here broaden the concept of self-organization in this context beyond these data and suggest that both epithelial cells and telocytes serve as sources of the organizing factor BMP2. Together, these data establish that in addition to the well-explored effects of BMP signaling in the epithelium^28,45^, its effects on the stroma extend its critical role in tissue homeostasis.

It should be noted that our model in its current form has several limitations. Firstly, it does not incorporate the immune cell compartment and future studies should explore the possibility of including immune cells. Secondly, its feasibility in human cells still needs to be explored. Additionally, we observed that crypt formation tended to be most pronounced towards the assembloid surface. Understanding the mechanisms responsible for this (e.g. hypoxic status across the assembloid) could yield new insights into cellular self-organization. Finally, our model will also enable investigations into the function and maturation of the enteric nervous system and endothelium, which can significantly impact gut homeostasis.

## Methods

### Mice

All procedures involving animals were approved by the institutional, local, and national legal authorities (LaGeSo Berlin, T-CH 0032/20) at the Charité Universitätsmedizin Berlin. C57BL/6 mice were obtained from Charles River Laboratory; *tg Act-DsRed*^46^, *tg Act-CFP*^47^, and *Rosa26-tdTomato*^48^ reporter mice were described previously. For lineage tracing of cells derived from AXIN2-expressing cells, *Axin2*^*CreERT2*^*/Rosa26-tdTomato* mice were generated by breeding *Axin2*^*CreErt2*^^49^ to *Rosa26-tdTomato* mice. *Bmpr1a*^*fl/fl*^ mice were obtained from the laboratory of Yuji Mishina^50^. To generate conditional KO mice with depletion of *Bmpr1a* in AXIN2^+^ cells, we bred *Bmpr1a*^*fl/fl*^ mice to *Axin2*^*CreERT2*^ mice. All animals were maintained in autoclaved micro-isolator cages and provided with sterile drinking water and chow ad libitum. The mice were bred at the animal care facility on a 12-h light/12-h dark cycle in a controlled temperature (22.5 ± 2.5 °C) and humidity (50 ± 5%) environment. Male 6 to 12-week-old mice were used for this study.

### Primary murine colon/stomach organoid culture

The mouse colon/stomach was dissected, opened longitudinally, and cut into 1 cm long pieces. Tissue was washed three times in 1x phosphate-buffered saline (PBS) (Gibco), followed by incubation for 20 min in 10 mM EDTA (Invitrogen)/PBS supplemented with 0.5 mM dithiothreitol (DTT) (Sigma) at 37 °C. Buffer was changed to ice-cold PBS, and the tube was vigorously shaken for 30 sec to isolate the colon crypts/gastric glands. The supernatant containing the crypts/glands was collected and centrifuged at 400*g* for 5 min at 4 °C, and the tissue fragments used for stromal cell isolation. The number of colonic crypts/gastric glands was determined, and 150 crypts/30 glands were resuspended and seeded in a 10 μl drop of Matrigel (Corning) (or Cultrex (R&D)) in a 24-well plate (three drops per well). Matrigel was polymerized at 37 °C for 15 min and then supplemented with 500 μl colon organoid medium: Advanced Dulbecco’s Modified Eagle’s Medium (DMEM)/F12 (Gibco) containing 10 mM HEPES (Gibco), 2 mM GlutaMAX (Gibco), 100 U/ml penicillin/streptomycin (Gibco), 1.25 mM N-acetylcysteine (Sigma), 1x B-27 (Gibco), 1x N2 (Gibco), 50 ng/ml mouse epidermal growth factor (EGF) (Invitrogen), and 0.5 μM A83-01 (Sigma), which was supplemented with 0.328 nM surrogate Wnt (sWnt, U-Protein Express), 3 μM CHIR-99021 (Selleckchem), 25% R-spondin 1 conditioned medium, and 100 ng/ml mouse noggin (PeproTech) for maintenance in the full medium (FM) condition (stomach organoid medium: Advanced DMEM/F12 supplemented with 10 mM HEPES, 2 mM GlutaMAX, 100 U/ml penicillin/streptomycin, 1.25 mM N-acetylcysteine, 1x B27, 1x N2, 50 ng/ml mouse EGF, 100 ng/ml mouse noggin, 100 ng/ml fibroblast growth factor 10, 10 nM gastrin, 10% R-spondin 1 conditioned medium, and 0.164 nM sWnt). 10 μM Y-27632 (Hoelzel) was added after the initial seeding and after passaging. The organoids were incubated at 37 °C, 5% CO_2_ in a humidified incubator. The medium was replaced every 1-2 days. Every 4–5 days (6 days for stomach organoids), the organoids were passaged as single cells generated by a 5-min incubation in TryplE (Gibco) at 37 °C. A total of 2500 cells were seeded per 10 μl Matrigel drop in a 24-well plate (three drops per well). The organoids were cultured for at least one passage before specific experiments.

Where indicated, sWnt, CHIR-99021, R-spondin 1, and noggin were withdrawn (-WCRN) for 48 h to induce organoid differentiation after two days of culture in FM. For BMP2 treatment, organoids were cultured in FM for two days followed by 48 h of culture with indicated concentrations of recombinant mouse BMP2 (R&D, 355-BM-010) and removal of noggin. To generate *Bmpr1a* KO organoids, 1.5 μM 4OH-tamoxifen (Sigma) was added to cultures from *Axin2*^*CreErt2*^*/Bmpr1a*^*fl/fl*^ mice for 24 h.

### Primary murine colon/stomach stromal cell culture

To isolate colon/stomach stromal cells, we further processed the tissue fragments from crypt/gland isolation. The tissue fragments were washed four times with ice-cold PBS and shaken after every wash to get rid of epithelium. When clean supernatant was obtained, the tissue fragments were cut into tiny pieces and incubated in calcium and magnesium-free HBSS (Gibco) containing Liberase TL (1 unit/ml; Roche) and DNase I (1 unit/ml; Invitrogen) at 37 °C for 1 h, with pipetting every 10 min. Every 20 min, the digested fraction was collected and put into ice-cold stromal cell medium containing Advanced DMEM/F12, 10% fetal bovine serum (FBS, Gibco), 100 U/ml penicillin/streptomycin, and 10 μM Y-27632. The collected fraction was filtered through a 70-μm cell strainer (Falcon) and centrifuged at 400 g for 5 min at 4 °C. The colon stromal cells were seeded on 12- or 6-well plates, or 75 cm^2^ flasks, and cultured in stromal cell medium.

For BMP2 or GREM1 treatments, stromal cells were cultured with recombinant mouse proteins: 50 ng/ml BMP2 or/and 500 ng/ml GREM1 (R&D, 956-GR-050) for 5 days, unless otherwise indicated.

To perform conventional epithelium-stroma co-culture, 4 × 10^4^ primary stromal cells were added to 2500 epithelial cells in a 10 μl Matrigel drop and cultured with organoid medium for 4 days before analysis.

### Reconstitution of colon/stomach assembloids

3-4 day-old colon/4-5 day-old stomach organoids were collected by physically pipetting with organoid harvesting solution (R&D) and put on ice for 30 min. When the Matrigel was depolymerized completely, the intact organoids were gently washed with Advanced DMEM/F-12 and allowed to settle by gravity (or centrifuging at 100*g* for 1 min at 4 °C). The supernatant was removed, and the organoids were resuspended in Matrigel. The primary colon/stomach stromal cells cultured in 75 cm^2^ flasks were treated with 5 ml TrypLE and incubated for 15 min at 37 °C. The dissociated cells were washed with Advanced DMEM/F-12 and centrifuged at 400*g* for 5 min at 4 °C. The cell number was determined, and cells were resuspended in Matrigel (1.5 × 10^5^ cells per 3.5 μl Matrigel). After being sprayed with 70% (vol/vol) ethanol, a 2.5 cm × 3.0 cm sheet of Parafilm was placed in a 100 mm petri dish, with several drops of PBS placed around the Parafilm to maintain humidity. A 2 μl Matrigel droplet containing stromal cells was placed on the Parafilm using a P10 pipette. Then 0.5 μl of organoids with 1.5 μl of stromal cells were immediately but slowly added into the droplet (4 μl Matrigel per assembloid in total). Ideally, organoids should be in the center of the droplet and the organoid number should be comparable. The petri dish containing 6 assembloids was covered with a lid and incubated at 37 °C for 10–15 min to solidify the gel. The assembloids were removed from the Parafilm using fine forceps and put into a 12-well plate (6–8 assembloids per well). Unless otherwise indicated, each well contained 1.5 ml organoid medium without sWnt, CHIR-99021, R-spondin 1, and noggin. The plate was put onto an orbital shaker (Edmund Buehler) at 170 rpm in the cell incubator. The medium was replaced every other day. Mature assembloids were collected for further analyses 4-5 days later (stomach assembloids: 6-7 days) unless indicated otherwise. Mature assembloids can be passaged as described for cell dissociation from assembloids in “Single-cell RNAsequencing” and reconstituted with fresh stromal cells.

To monitor assembloid growth, the epithelial cells from *tg Act-DsRed* mice were assembled with the stromal cells from *tg Act-CFP* mice. To visualize AXIN2^+^ cells and their immediate progeny, the assembloids were generated with epithelial cells from *Axin2*^*CreERT2*^*/Rosa26-tdTomato* mice and treated with 1.5 μM 4OH-tamoxifen for 24 h before collecting. *Bmpr1a* KO organoids were assembled with WT stromal cells to make epithelium-specific *Bmpr1a* KO (*Bmpr1a*^*ΔEPI*^) assembloids. To induce knock-out of *Bmpr1a*, the stromal cells from *Bmpr1a*^*fl/fl*^ mice were treated with 4 μM TAT-CRE Recombinase (Millipore, SCR508) for 24 h. The KO stromal cells were expanded, harvested, and assembled with WT organoids to make mesenchyme-specific *Bmpr1a* KO (*Bmpr1a*^*ΔMES*^) assembloids.

### Reconstitution of colon tumor assembloids

To induce colitis-associated tumors, mice were injected intraperitoneally with 9.5 mg/kg azoxymethane (AOM, Sigma, A5486). Five days later, mice received 2% dextran sodium sulfate (DSS, MP Biomedical, 160110) in drinking water over six days. Mice were monitored for three months before analyses. The colon was dissected and opened longitudinally for the assessment of tumors. To set up organoid cultures, tumor tissue was dissected free, and single tumors cut free from the colon using scissors, minced into small pieces with a scalpel, and incubated in Advanced DMEM/F12 + Liberase TL (1 unit/ml) at 37 °C for 20 min with repeated trituration. Cell clusters were centrifuged, resuspended in TrypLE, and incubated at 37 °C for 10 min, followed by a wash in 0.1% BSA/PBS. Single cells were then seeded in Matrigel and cultured with full organoid medium. sWnt and CHIR-99021 were withdrawn to select Wnt-independent organoids. These Wnt-independent organoids were then used to generate colon tumor assembloids.

### Single-cell RNA-sequencing

For stromal/epithelial cell dissociation from assembloids, samples were incubated in calcium and magnesium-free HBSS containing Liberase TL (1 unit/ml) and DNase I (1 unit/ml) at 37 °C for 1 h, with pipetting every 10 min. Every 20 min, the digested fraction was collected and put into ice-cold medium containing Advanced DMEM/F12, 10% FBS, 10% penicillin/streptomycin, and 10 μM Y-27632. The collected fraction was filtered through a 70-μm cell strainer and centrifuged at 400 g for 5 min at 4 °C. Cell clusters were incubated with TrypLE for 5 min at 37 °C and filtered through a 40-μm cell strainer. For epithelial cell dissociation from colon tissue, samples were processed as described for “Primary murine colon/stomach organoid culture”. Colonic crypts were collected and incubated with TrypLE for 5 min at 37 °C and filtered through a 40-μm cell strainer. For epithelial cell dissociation from organoids, samples were incubated with TrypLE for 5 min at 37 °C and filtered through a 40-μm cell strainer. Each sample was run with Chromium single cell kits (10x Genomics), following the manufacturer’s instructions.

For the analysis of stromal cells, a total of 16242 EpCAM-negative cells from assembloids (derived from two C57BL/6 mice) were analyzed. Seurat v3 R package^51^ was used for the whole pipeline, using a cut-off of at least 500 genes in a single cell and a maximum of 10% mitochondrial reads. Normalization was carried out using the SCTransform function, including a mitochondrial percentage regression. Integration was performed with CCA as implemented in Seurat to eliminate the batch effect of the two samples. A Uniform Manifold Approximation and Projection (UMAP) analysis was applied using the first 30 PCA dimensions. To conduct cluster identification according to previous publications, we adjusted the resolution value (value = 0.2) when performing the Seurat FindClusters function, and the cluster number was then determined automatically. We then evaluated each cluster in detail and annotated the clusters based on the gene expression patterns. For the dot plot presentation, we represented the expression of genes in each cluster as the scaled average level of expression of those cells and the proportion of cells that express those genes.

We reanalyzed previously published scRNA-Seq data from healthy in vivo mouse colon stromal cells from GSE114374^2^ (*n* = 3 samples) and from GSE172261^23^ (*n* = 6 samples) to compare stromal cell populations from the assembloid data. We filtered and preprocessed both data sets with SCTransform and integrated them using CCA. We then identified in vivo stromal clusters by label transfer from GSE172261 and published marker gene expression, followed by identification of cluster markers using Seurat’s FindAllMarkers. We performed Gene Set Variation Analysis using the cluster marker genes of colon stromal cells as signatures and computed per-cell scores for each cluster signature on assembloid stroma data using the R package GSVA 1.48.0^52^ with standard parameters. We used the R package pheatmap 1.0.12 (https://CRAN.R-project.org/package=pheatmap) to analyze the average expression of genes in different stromal clusters in the colon tissue.

scRNA-Seq of epithelial cells from C57BL/6 mouse colon tissue (*n* = 2 samples), assembloids (*n* = 2 samples), and organoids (*n* = 2 samples) resulted in 12,357, 16,787, and 13,352 cells after filtering. Cells were preprocessed using SCTransform and integrated individually using Harmony 0.1.1^53^ within each group, adjusting for batch effects. Further data analyses were performed as described for scRNA-Seq of assembloid stroma.

### Histology and imaging

Organoids/assembloids were transferred to 4% paraformaldehyde (PFA) and fixed for 2 h at room temperature (assembloids were fixed overnight at 4 °C), followed by incubation in 0.1% BSA/PBS for at least 30 min, embedding in 2% agarose, dehydration, and embedding in paraffin. 5 μm sections were deparaffinized and rehydrated, followed by antigen retrieval in citrate buffer. Non-specific antibody binding was blocked by incubation in 0.1% Tween 20/PBS supplemented with 5% FBS and 1% BSA for 1 h, followed by overnight incubation with primary antibodies. The next day, they were incubated with secondary antibodies for 2 h at room temperature and counterstained with DAPI (Sigma, 1:300). For whole-mount staining of assembloids/colon tissue, they were fixed in 4% PFA for one hour (two hours for tissue) at room temperature, then washed with PBS followed by overnight incubation in permeabilization buffer (PBS with 3% BSA, 1% saponin, 2% Triton X-100, and 0.02% sodium azide) substituted with DAPI/fluorophore-conjugated antibodies. The following antibodies were used: Rabbit anti-KI67 (Cell Signaling Technology, 9129S, clone D3B5, 1:100), rabbit anti-keratin 20 (Cell Signaling Technology, 13063S, clone D9Z1Z, 1:100), rabbit anti-MUC2 C3^54,55^ (a gift from Prof. Gunnar C. Hansson, 1:1000), rabbit anti-synaptophysin (Abcam, ab178412, clone EPR1097-2, 1:100), rabbit anti-active YAP1 (Abcam, ab205270, clone EPR19812, 1:200), mouse anti-E-cadherin (BD, 610181, clone 36, 1:200), goat anti-αSMA (Abcam, ab5694, 1:100); rabbit anti-vimentin (Cell Signaling Technology, 5741S, clone D21H3, 1:100); rabbit anti-CD31 (Cell Signaling Technology, 77699S, clone D8V9E, 1:100); rabbit anti-β3-tubulin (Cell Signaling Technology, 5568S, clone D71G9, 1:100); Alexa Fluor 647-conjugated phalloidin (Life Technologies, A22287, 1:100); mouse anti-MUC5AC (Invitrogen, 12178, clone 45M1, 1:100); Alexa Fluor 647-conjugated lectin GSII (Thermo Scientific, L32451, 1:100); rabbit anti-cleaved caspase 3 (Cell Signaling Technology, 9661S, clone Asp175, 1:100). AlexaFluor 488 donkey anti-mouse IgG (Jackson Immunoresearch, 715-546-150, 1:250); Cy3 donkey anti-rabbit IgG (Jackson Immunoresearch, 711-166-152, 1:250); AlexaFluor 647 donkey anti-goat IgG (Jackson Immunoresearch, 705-605-003, 1:250). Immunofluorescence images were acquired with the confocal laser scanning microscope TCS SP8 (Leica) and Observer 7 microscope (Zeiss). Live cell imaging was done on the EVOS M5000 imaging system (Invitrogen). Images were collected and analyzed with Leica Application Suite X 3.5.6.21594 (LAS X, Leica), ZEN 3.4 (Zeiss), and ImageJ 2.0.0 (Fiji).

### Tissue processing

For paraffin embedding, colon tissue was flushed with PBS and fixed in 4% PFA overnight at 4 °C. Paraffin embedding and sectioning, as well as hematoxylin and eosin (H&E) staining, were performed by the Charité Core Unit Immunopathology for Experimental Models.

### Single-molecule RNA in situ hybridization

Five micrometer thick assembloid/colon tissue sections were used for single-molecule in situ hybridization. RNA in situ detection was performed with the RNAscope Red Detection Kit (Advanced Cell Diagnostics, 322360) or Duplex Detection Kit (Advanced Cell Diagnostics, 322500) according to the manufacturer’s protocol. According to the manufacturer’s instructions, positive and negative control probes were used for each experiment. For individual genes, the following probe target regions were used: for *Rspo3* 731–2164, for *Foxl1* 954–1931, for *Bmp2* 854–2060, for *Grem1* 398–1359, for *Wnt5a* 200–1431, for *Id1* 2–705, for *Pdgfra* 223–1161.

### Flow cytometry

The colon stromal cells were extracted from culture plates for CD34^+^ cell proportion analysis after BMP2 treatment. Sub-populations of cells were stained using antibodies against cell surface markers, including PE-conjugated rat anti-CD326 (EpCAM) (Miltenyi Biotec, 130-117-779, clone caa7-9G8, 1:200), APC-Cy7-conjugated rat anti-CD45 (BD, 557659, clone 30-F11, 1:200), APC-conjugated rat anti-CD31 (BD, 551262, clone MEC 13.3, 1:200), and FITC-conjugated rat anti-CD34 (BD, 560238, clone RAM34, 1:100), and compensation control (UltraComp eBeads, Invitrogen, 01-2222-42). Live/dead staining was performed by incubating cells with fixable viability stain FVS450 (BD, 562247, 1:1000) according to the manufacturer’s protocol. Flow cytometry experiments were performed on a Cytek Aurora 3 laser spectral cytometer (CyTek Biosciences). Data were analyzed with FCS Express 7 (De Novo) software.

For the experiment of BMP2 treatment of CD34^+^ cells, stromal cells were isolated from mouse colon tissue as described above. Following appropriate antibody staining (CD326 (EpCAM) PE (1:200), CD45 APC-Cy7 (1:200), CD31 APC (1:200), CD34 FITC (1:100), FVS450 (1:1000)) and compensation control, samples were sorted into CD34^+^ (live, EpCAM^-^ CD45^-^ CD31^-^ CD34^+^) or CD34^-^ (live, EpCAM^-^ CD45^-^ CD31^-^ CD34^-^) populations in sterile condition using a FACS Aria III (BD).

### RNA isolation and RT-PCR

Cell medium was removed following retrieval of cells from cultures, and RNA was isolated using the NucleoSpin RNA isolation kit (Macherey & Nagel, 740955) according to the manufacturer’s instructions. cDNA was generated using the iScript cDNA synthesis kit (Bio-Rad, 1708891), and qPCR was performed with SYBR-green (Thermo Fisher, A25741) and the QuantStudio Real-Time PCR System (Thermo Fisher). The data were collected with QuantStudio 3 Real-Time PCR Software v1.7.1. Fold-change expression was determined following the delta C_t_ method and normalized to *Gapdh*.

The following mouse primers were used: *Gapdh* forward 5′- TCACCATCTTCCAGGAGCG-3′, reverse 5′-AAGCAGTTGGTGGTGCAGG-3′; *Bmp2* forward 5′- GACTGCGGTCTCCTAAAGGTCG-3′, reverse 5′-CTGGGGAAGCAGCAACACTA-3′; *Id1* forward 5′-GCTCTACGACATGAACGGCT-3′, reverse 5′-AACACATGCCGCCTCGG-3′; *Lgr5* forward 5′-CCTACTCGAAGACTTACCCAGT-3′, reverse 5′-GCATTGGGGTGAATGATAGCA-3′; *Krt20* forward 5′-GTCCCACCTCAGCATGAAAGA-3′, reverse 5′-TCTGGCGTTCTGTGTCACTC-3′; *Foxl1* forward 5′-TCATCATGGATCGCTTCCCG-3′, reverse 5′-CCTCTTCCTGCGCCGATAAT-3′; *Wnt5a* forward 5′-ACGCTATACCAACTCCTCTGC-3′, reverse 5′-AATATTCCAATGGGCTTCTTCATGG-3′; *Cd34* forward 5′-GGCCAATAGCACAGAACTTCC-3′, reverse 5′-CCCAACAGCCATCAAGGTTC-3′; *Mgp* forward 5′-CCGAGACACCATGAAGAGCC-3′, reverse 5′-GTTGCGTTCCTGGACTCTCTT-3′; *Rspo3* forward 5′-TTGACAGTTGCCCAGAAGGG-3′, reverse 5′-CTGGCCTCACAGTGTACAATACT-3′; *Grem1* forward 5′-AAGTGACAGAATGAATCGCACC-3′, reverse 5′-CCTCAGCTGTTGGCAGTAGG-3′; *Wnt4* forward 5′-CGAGCAATTGGCTGTACCTG-3′, reverse 5′-GGGAGTCCAGTGTGGAACAG-3′; *Gata3* forward 5′-GCTCCTTGCTACTCAGGTGAT-3′, reverse 5′-GGAGGGAGAGAGGAATCCGA-3′; *Smad6* forward 5′-CCCTATTCTCGGCTGTCTCC-3′, reverse 5′-CTGGCATC TGAGAATTCACCC-3′; *Gpr20* forward 5′-GAAGGCACTGTCTGTGGGTC-3′, reverse 5′-TCCCCGAGCCCGCTCTT-3′; *Rab3b* forward 5′-GGGAACCCAACCCATCTTCA-3′, reverse 5′-AGTCACTGAAGCCATCTCGGA-3′; *Sox6* forward 5′- GCCAGGTGATAACTACCCCG −3′, reverse 5′- TGAGCGGCATAGAGCTGAAG −3′; *C3* forward 5′-TTCCTTCACTATGGGACCAGC-3′, reverse 5′TCCTTACTGGCTGGAATCTTGA-3′; *Il33* forward 5′-CCTCCCTGAGTACATAACATGACC-3′, reverse 5′-GTAGTAGCACCTGGTCTTGCTCTT-3′; *Sfrp1* forward 5′-CAAGCGAGTTTGCACTGAGG-3′, reverse 5′- CCCCAGCTTCAAGGGTTTCT −3′; *Angptl7* forward 5′-TAGCTACTTTGCGTTGGGCA-3′, reverse 5′-ACCAGTAGCCACCTTTTCGG-3′; *Cfb* forward 5′-TCAAGAATGGGGACAAGAAAGC-3′, reverse 5′- CTTTGCATGTGTTGGGGTCA-3′; *Il6* forward 5′-CGGCCTTCCCTACTTCACAA-3′, reverse 5′-TTGCCATTGCACAACTCTTTTC-3′.

### Statistics and reproducibility

Data are expressed as mean ± SEM. *P* < 0.05 was considered significant. Student’s *t*-test (two-tailed) was used to determine differences between two groups. One-way ANOVA followed by respective post-hoc analysis (Tukey’s test) was used for statistical comparison of more than two groups. Data are displayed for at least three independent biological replicates, except for the scRNA-seq of stromal cells from assembloids and epithelial cells from assembloids, organoids, and colon tissue, for which two biological replicates per group were used. In Figs. 1b–e, h; 2a, b, f; 3d, e; 4a, h; Supplementary Figs. 1a, c-h; 2c, d; 4a–c; 5a; 8a, b, e–g, at least three independent experiments were performed with similar results. In Figs. 1g, 3g, 4c; Supplementary Fig. 2a, b, the average percentage of specific epithelial cells from three biological replicates was analyzed. In Figs. 3c, h, 4d–f; Supplementary Fig. 7a, e, signal areas in random fields of view from three biological replicates were analyzed. In Supplementary Fig. 8c, d, all organoids in three Matrigel drops each group from three biological replicates were analyzed. GraphPad Prism 8, R 4.2.2, and RStudio 2021.09.1 were used for data visualization and statistical analysis.

### Reporting summary

Further information on research design is available in the Nature Portfolio Reporting Summary linked to this article.

## Supplementary information

Supplementary Information

Reporting Summary

## Data Availability

All scRNAseq data generated in this study have been deposited in the National Centre for Biotechnology, Gene Expression Information Omnibus (GEO) under accession code GSE231716. The previously published scRNAseq data are available in the GEO under accession code GSE114374 and GSE172261. Source data are provided with this paper.

## Source data

Source Data

## Code availability

All computational codes can be accessed under https://github.com/Sigal-Lab/Lin_Colon_Assembloids.
