# Supplementary material for: Establishment of gastrointestinal assembloids to study the interplay between epithelial crypts and their mesenchymal niche
Source: Nat Commun. 2023 May 25;14:3025. doi: 10.1038/s41467-023-38780-3 (PMC10212920; doi:10.1038/s41467-023-38780-3)
Supplement: Supplementary file 1 — Supplementary Information [file 41467_2023_38780_MOESM1_ESM.pdf]

Supplementary Information

Establishment of gastrointestinal assembloids to study the interplay between epithelial crypts and their mesenchymal niche

Manqiang Lin, Kimberly Hartl, Julian Heuberger, Giulia Beccaceci, Hilmar Berger, Hao Li, Lichao Liu, Stefanie Muellerke, Thomas Conrad, Felix Heymann, Andrew Woehler, Frank Tacke, Nikolaus Rajewsky, Michael Sigal

This PDF file includes:

Supplementary Figures 1-8

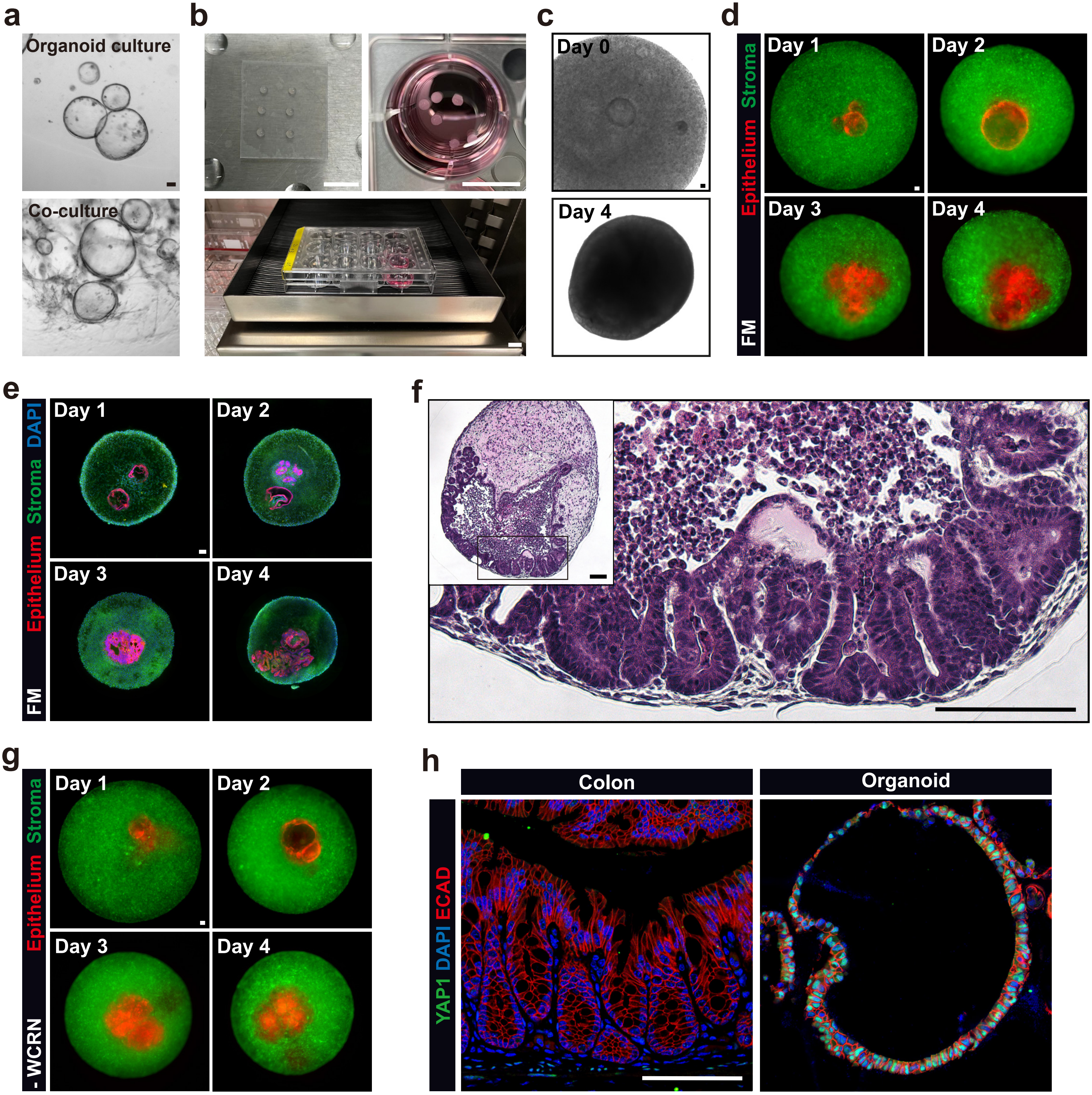
a
Organoid culture
Co-culture
b
c
Day 0
Day 4
d
Epithelium Stroma
FM
Day 1
Day 2
Day 3
Day 4
e
Epithelium Stroma DAPI
FM
Day 1
Day 2
Day 3
Day 4
f
g
Epithelium Stroma
- WCRN
Day 1
Day 2
Day 3
Day 4
h
Colon
Organoid
YAP1 DAPI ECAD

Supplementary Figure 1. Establishment of colon assembloid culture system. (**a**) Bright-field images of conventional colon organoid culture and organoids co-cultured with primary colon stromal cells. Scale bar: 100 µm. (**b**) Upper left panel: droplets of assembloids were placed on the Parafilm. Upper right panel: assembloids were cultured in a 12-well plate. Lower panel: the culture plate was placed on an orbital shaker. Scale bars: 1 cm. (**c**) Bright-field images of assembloids on day 0 and day 4. Scale bar: 100 µm. (**d**) Live fluorescent images and (**e**) confocal microscopy images of assembloids cultured in full medium (FM) for 1 - 4 days. Epithelium was derived from *tg Act-DsRed* mice; stroma was derived from *tg Act-CFP* mice. Scale bars: 100 µm. (**f**) Hematoxylin and eosin (H&E) staining images of assembloids cultured in FM for 4 days. Scale bar: 100 µm. (**g**) Live fluorescent images of assembloids cultured in medium without sWnt, R-spondin 1, CHIR99021, or noggin (-WCRN) for 1 - 4 days. Epithelium was derived from *tg Act-DsRed* mice; stroma was derived from *tg Act-CFP* mice. Scale bar: 100 µm. (**h**) Immunofluorescence images of colon tissue and organoids stained for active YAP1. Scale bar: 100 µm. Images are representative of at least three biological replicates.

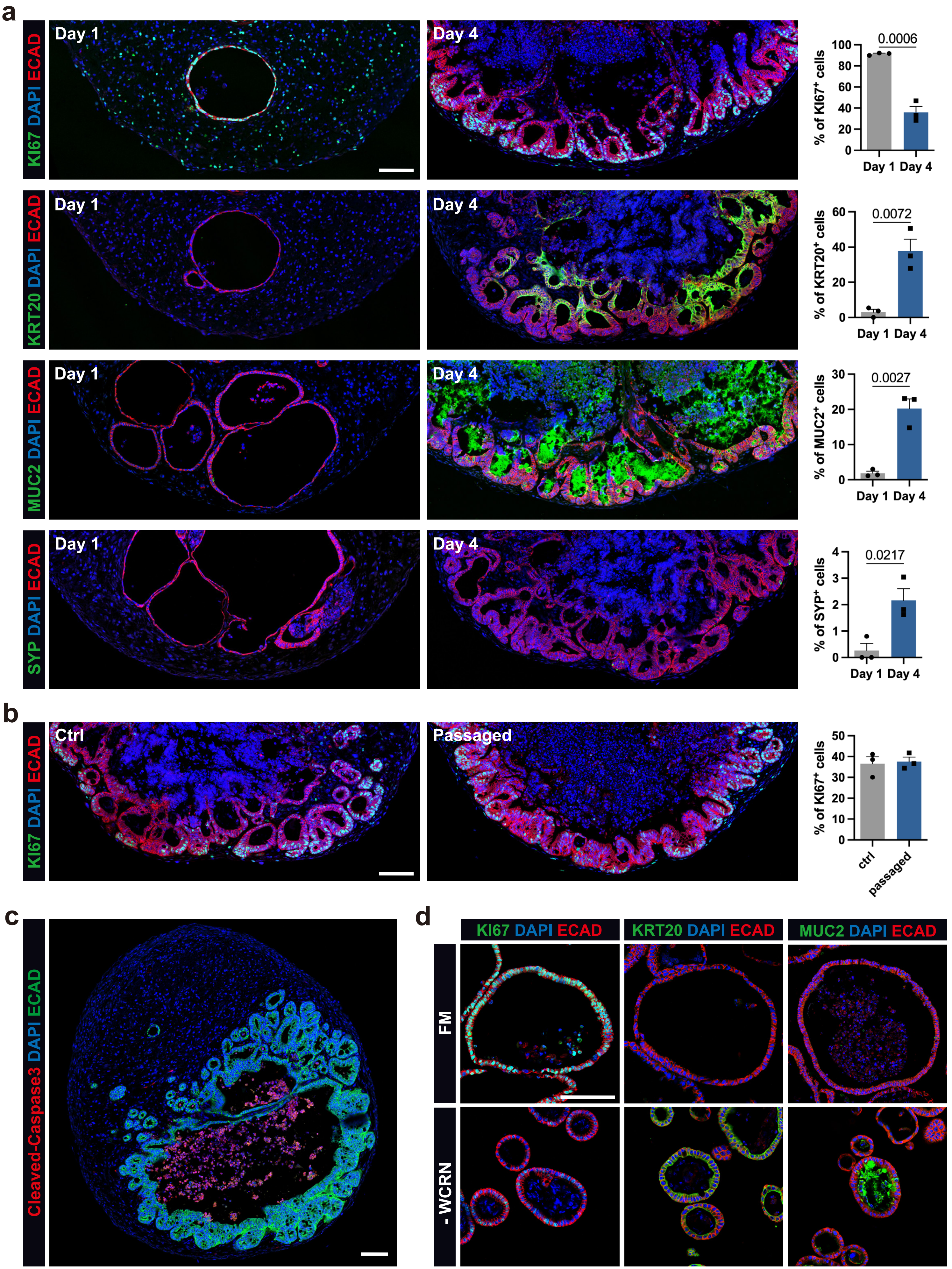
a
KI67 DAPI ECAD
Day 1
Day 4
0.0006
% of KI67+ cells
100
80
60
40
20
0
Day 1 Day 4
KRT20 DAPI ECAD
Day 1
Day 4
0.0072
% of KRT20+ cells
60
40
20
0
Day 1 Day 4
MUC2 DAPI ECAD
Day 1
Day 4
0.0027
% of MUC2+ cells
30
20
10
0
Day 1 Day 4
SYP DAPI ECAD
Day 1
Day 4
0.0217
% of SYP+ cells
4
3
2
1
0
Day 1 Day 4
b
KI67 DAPI ECAD
Ctrl
Passaged
% of KI67+ cells
50
40
30
20
10
0
ctrl
passaged
c
Cleaved-Caspase3 DAPI ECAD
d
KI67 DAPI ECAD
KRT20 DAPI ECAD
MUC2 DAPI ECAD
FM
- WCRN

Supplementary Figure 2. Epithelial cell organization in assembloids. (**a**) Immunofluorescence images and quantification of colon assembloids on days 1 and 4 labeled with markers for proliferative cells (KI67), colonocytes (KRT20), goblet cells (MUC2), and enteroendocrine cells (SYP) (*n* = 3 biological replicates per group). (**b**) Immunofluorescence images and quantification of control and passaged assembloids labeled for proliferative cells (KI67) (*n* = 3 biological replicates per group). (**c**) Immunofluorescence images of colon assembloids stained for cleaved-caspase 3. (**d**) Immunofluorescence images of colon organoids cultured in full medium (FM) or in medium without sWnt, R-spondin1, CHIR99021, or noggin (-WCRN), stained for KI67, KRT20, and MUC2. Scale bars: 100 μm. Data are presented as mean ± SEM. Statistical analyses were performed using Student's *t*-test (two-tailed) for **a** and **b**. Images are representative of at least three biological replicates. Source data are provided as a Source Data file.

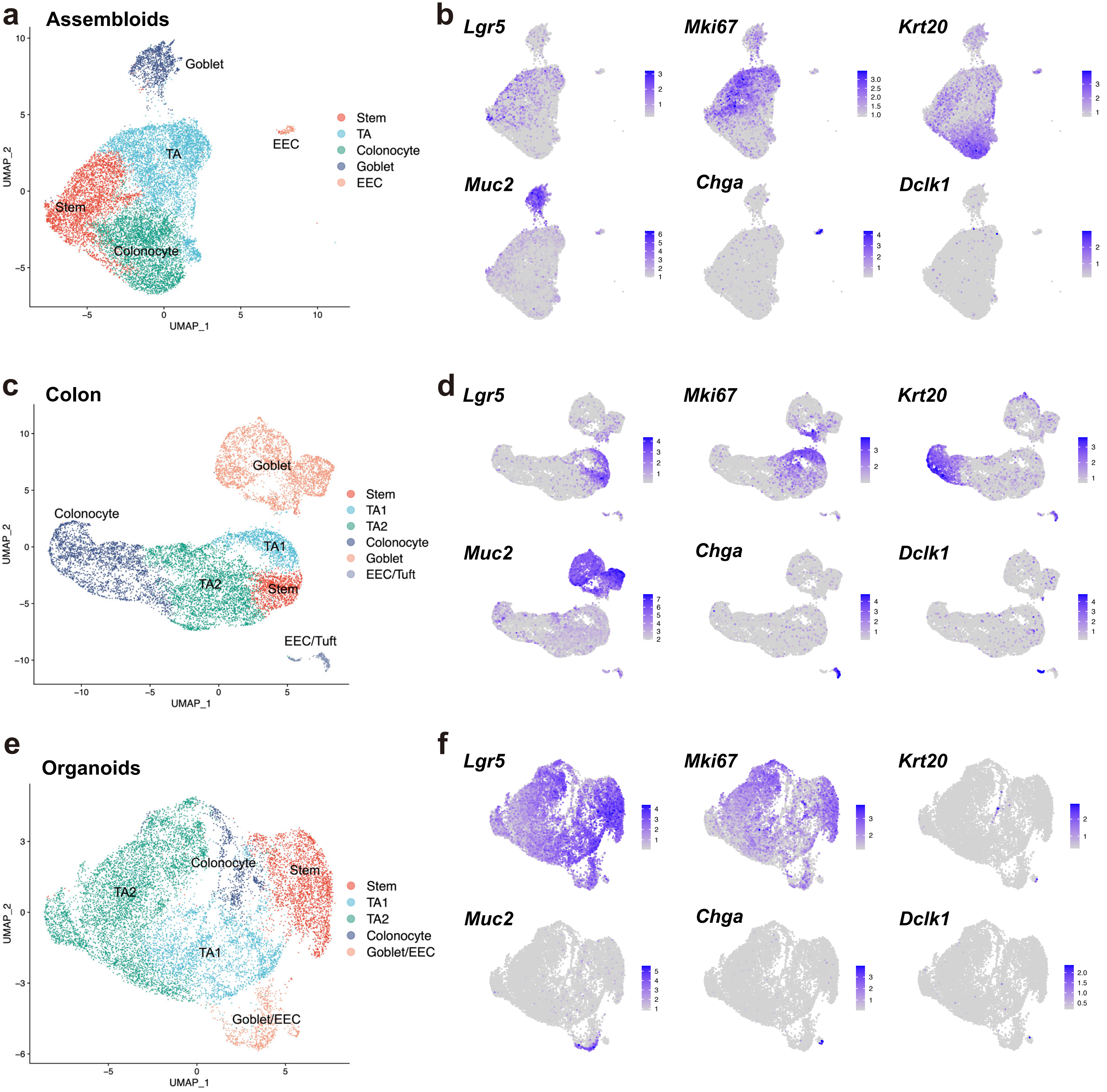

a
Assembloids
Goblet
TA
EEC
Stem
Colonocyte
UMAP_2
UMAP_1
Stem
TA
Colonocyte
Goblet
EEC
b
Lgr5
Mki67
Krt20
Muc2
Chga
Dclk1
c
Colon
Goblet
Colonocyte
TA1
TA2
Stem
EEC/Tuft
UMAP_2
UMAP_1
Stem
TA1
TA2
Colonocyte
Goblet
EEC/Tuft
d
Lgr5
Mki67
Krt20
Muc2
Chga
Dclk1
e
Organoids
Colonocyte
Stem
TA2
TA1
Goblet/EEC
UMAP_2
UMAP_1
Stem
TA1
TA2
Colonocyte
Goblet/EEC
f
Lgr5
Mki67
Krt20
Muc2
Chga
Dclk1

Supplementary Figure 3. Epithelial cell complexity of colon assembloids, tissue, and organoids. (**a**) UMAP plot of scRNA-seq dataset from epithelial cells of colon assembloids. (**b**) UMAP expression plots of stem cell marker gene *Lgr5*, proliferative cell marker *Mki67*, colonocyte marker *Krt20*, goblet cell marker *Muc2*, enteroendocrine cell marker *Chga*, tuft cell marker *Dclk1* in the assembloid dataset. Cells colored by normalized expression of indicated genes. (**c**) UMAP plot of scRNA-seq dataset from *in vivo* colon epithelial cells. (**d**) UMAP expression plots of marker genes of different types of epithelial cells in the dataset of colon tissue. Cells colored by normalized expression of indicated genes. (**c**) UMAP plot of scRNA-seq dataset from epithelial cells of colon organoids. (**d**) UMAP expression plots of marker genes of different types of epithelial cells in the dataset of colon organoids. Cells colored by normalized expression of indicated genes. TA, transit-amplifying cell; EEC, enteroendocrine cell. scRNA-seq data are from two biological replicates per group.

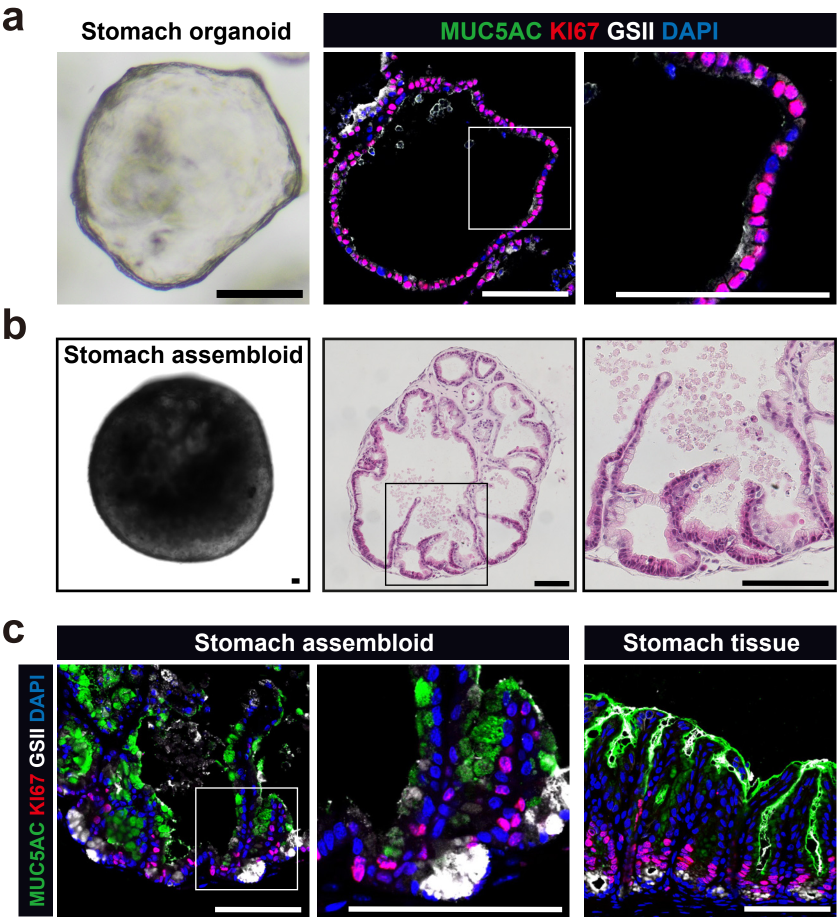
a
Stomach organoid
MUC5AC KI67 GSII DAPI
b
Stomach assembloid
c
Stomach assembloid
Stomach tissue
MUC5AC KI67 GSII DAPI

Supplementary Figure 4. Establishment of stomach assembloid culture system. (**a**) Bright-field image of conventional stomach organoid culture and immunofluorescence images of stomach organoids cultured in full medium, stained for pit mucous cells (MUC5AC), proliferative cells (KI67), and base secretory cells (GSII). (**b**) Bright-field image and hematoxylin and eosin (H&E) staining of murine stomach assembloids (cultured for 6 days in medium without Wnt surrogate, R-spondin1, or noggin). (**c**) Immunofluorescence images of a stomach assembloid and stomach tissue stained for MUC5AC, KI67, and GSII. Scale bars: 100 μm. Images are representative of at least three biological replicates.

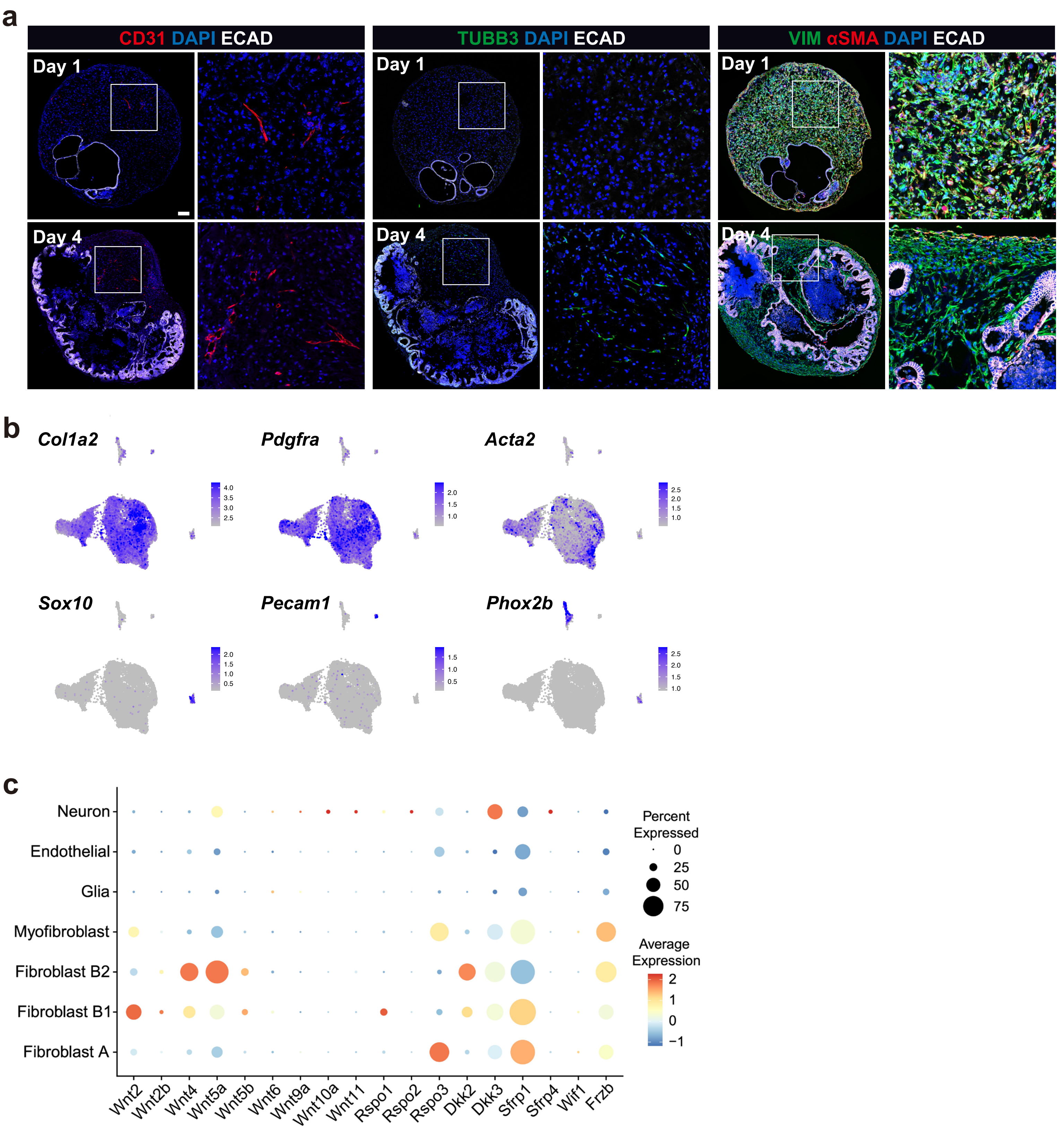
a
CD31 DAPI ECAD
TUBB3 DAPI ECAD
VIM αSMA DAPI ECAD
Day 1
Day 4
b
Col1a2
Pdgfra
Acta2
Sox10
Pecam1
Phox2b
c
Neuron
Endothelial
Glia
Myofibroblast
Fibroblast B2
Fibroblast B1
Fibroblast A
Wnt2
Wnt2b
Wnt4
Wnt5a
Wnt5b
Wnt6
Wnt9a
Wnt10a
Wnt11
Rspo1
Rspo2
Rspo3
Dkk2
Dkk3
Sfrp1
Sfrp4
Wif1
Frzb
Percent Expressed
0
25
50
75
Average Expression
2
1
0
−1

Supplementary Figure 5. The stromal compartment in colon assembloids. (**a**) Immunofluorescence images of colon assembloids (day 1 and day 4) stained for endothelial cells (CD31), neuronal cells (TUBB3), fibroblast (VIM), and myofibroblast (αSMA). Scale bar: 100 μm. (**b**) UMAP expression plots of known marker genes against stromal cell clusters in the assembloid dataset. Cells colored by normalized expression of indicated marker genes. (**c**) Dot plot showing expression of Wnt signaling molecules in identified clusters in assembloids. Circle size represents the within-cluster probability of gene detection. Fill color represents the normalized average expression level. Images are representative of at least three biological replicates. scRNA-seq data are from two biological replicates.

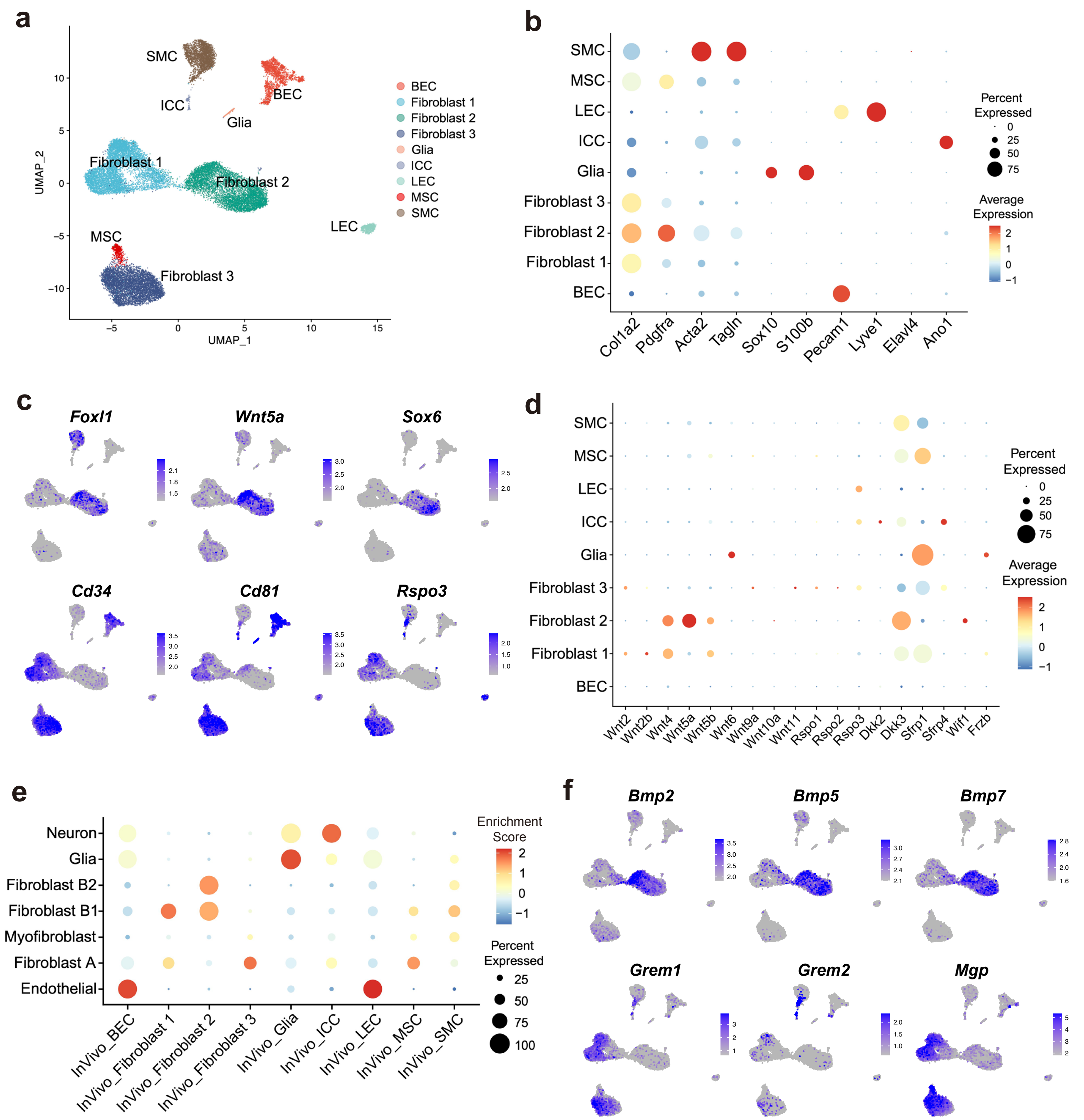
a
SMC
ICC
Glia
BEC
Fibroblast 1
Fibroblast 2
MSC
LEC
Fibroblast 3
UMAP_1
UMAP_2
BEC
Fibroblast 1
Fibroblast 2
Fibroblast 3
Glia
ICC
LEC
MSC
SMC
b
Col1a2
Pdgfra
Acta2
Tagln
Sox10
S100b
Pecam1
Lyve1
Elavl4
Ano1
Percent Expressed
Average Expression
c
Foxl1
Wnt5a
Sox6
Cd34
Cd81
Rspo3
d
Wnt2
Wnt2b
Wnt4
Wnt5a
Wnt5b
Wnt6
Wnt9a
Wnt10a
Wnt11
Rspo1
Rspo2
Rspo3
Dkk2
Dkk3
Sfrp1
Sfrp4
Wif1
Frzb
e
Neuron
Glia
Fibroblast B2
Fibroblast B1
Myofibroblast
Fibroblast A
Endothelial
InVivo_BEC
InVivo_Fibroblast 1
InVivo_Fibroblast 2
InVivo_Fibroblast 3
InVivo_Glia
InVivo_ICC
InVivo_LEC
InVivo_MSC
InVivo_SMC
Enrichment Score
Percent Expressed
f
Bmp2
Bmp5
Bmp7
Grem1
Grem2
Mgp

Supplementary Figure 6. The stromal compartment in colon tissue. (**a**) UMAP plot of scRNA-seq dataset from *in vivo* colon stromal cells. (**b**) Dot plot showing expression of known marker genes against detected stromal clusters in colon tissue identified by scRNA-seq. Circle size represents the within-cluster probability of gene detection. Fill color represents the normalized average expression level. (**c**) UMAP expression plots of telocyte marker genes (*Foxl1*, *Wnt5a*, and *Sox6*) and trophocyte marker genes (*Cd34*, *Cd81*, and *Rspo3*) in the colon. Cells colored by normalized expression of indicated marker genes. (**d**) Dot plot showing expression of Wnt signaling molecules in identified clusters in colon tissue. Circle size represents the within-cluster probability of gene detection. Fill color represents the normalized average expression level. (**e**) Gene Set Variation Analysis comparing stromal cluster gene signatures between colon tissue and assembloids. Circle size represents the within-cluster probability of gene signature detection. Fill color represents the normalized average enrichment score. (**f**) UMAP expression plots of BMP ligand genes (*Bmp2*, *Bmp5*, and *Bmp7*) and BMP antagonist genes (*Grem1*, *Grem2*, and *Mgp*) for stromal cells in colon tissue. BEC, blood endothelial cell; ICC, interstitial cell of Cajal; LEC, lymphatic endothelial cell; MSC, mesenchymal stem cell; SMC, smooth muscle cell. scRNA-Seq data of *in vivo* mouse colon stromal cells are from published datasets GSE114374 (three biological replicates) and GSE172261 (six biological replicates).

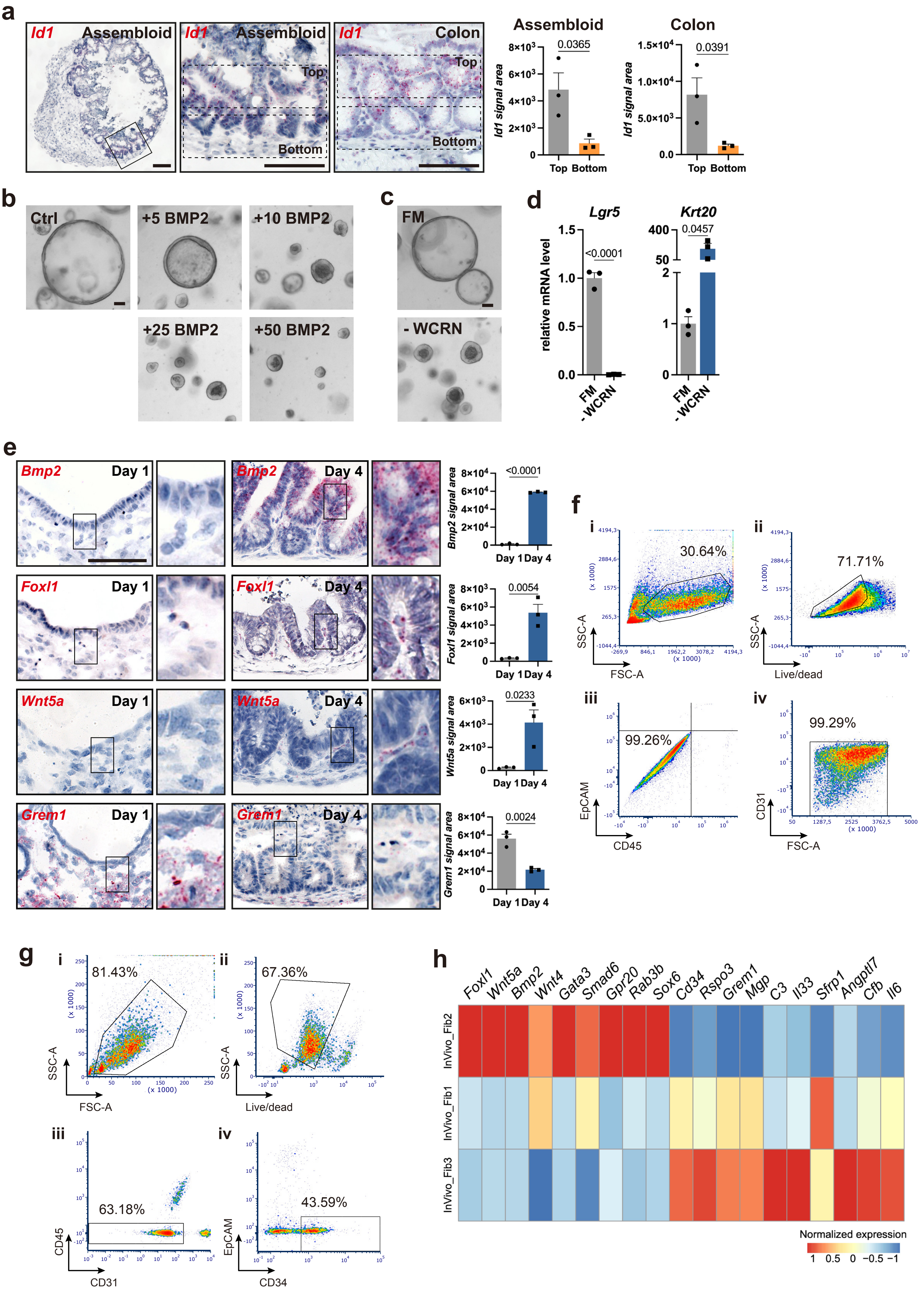
a
Id1 Assembloid
Id1 Assembloid
Top
Bottom
Id1 Colon
Top
Bottom
Assembloid
Id1 signal area
0.0365
Top Bottom
Colon
0.0391
Top Bottom
b
Ctrl
+5 BMP2
+10 BMP2
+25 BMP2
+50 BMP2
c
FM
- WCRN
d
Lgr5
Krt20
relative mRNA level
<0.0001
0.0457
FM
- WCRN
e
Bmp2 Day 1
Bmp2 Day 4
Foxl1 Day 1
Foxl1 Day 4
Wnt5a Day 1
Wnt5a Day 4
Grem1 Day 1
Grem1 Day 4
Bmp2 signal area
<0.0001
Foxl1 signal area
0.0054
Wnt5a signal area
0.0233
Grem1 signal area
0.0024
Day 1 Day 4
f
i 30.64%
SSC-A
FSC-A
ii 71.71%
Live/dead
iii 99.26%
EpCAM
CD45
iv 99.29%
CD31
FSC-A
g
i 81.43%
SSC-A
FSC-A
ii 67.36%
Live/dead
iii 63.18%
CD45
CD31
iv 43.59%
EpCAM
CD34
h
Foxl1 Wnt5a Bmp2 Wnt4 Gata3 Smad6 Gpr20 Rab3b Sox6 Cd34 Rspo3 Grem1 Mgp C3 Il33 Sfrp1 Angptl7 Cfb Il6
InVivo_Fib2
InVivo_Fib1
InVivo_Fib3
Normalized expression
1 0.5 0 −0.5 −1

Supplementary Figure 7. The effects of BMP signaling on colon epithelial and stromal cells. (**a**) Single-molecule in situ hybridization (sm-ISH) showing localization of *Id1* in assembloids and colon tissue; quantification for *Id1* signal area divided into top and bottom in the crypt (*n* = 3 biological replicates per group). (**b**) Bright-field images of colon organoids cultured in full medium (ctrl) or exposed to the indicated BMP2 concentrations (without noggin). (**c**) Bright-field images of colon organoids cultured in full medium (FM) or in medium without sWnt, R-spondin 1, CHIR99021, or noggin (-WCRN). (**d**) qPCR for expression of indicated genes from organoids cultured in FM or -WCRN medium (*n* = 3 biological replicates per group). (**e**) sm-ISH images and quantification of expression of *Bmp2*, *Foxl1*, *Wnt5a*, and *Grem1* in assembloids on days 1 and 4 (*n* = 3 biological replicates per group). (**f**) Representative flow cytometry gating strategies for analyses of alive ($FVS450^-$) EpCAM $(PE)^-$ CD45 (APC-$Cy7)^-$ CD31 $(APC)^-$ primary murine colonic stromal subsets. Cells were gated with forward and sideward scatter (FSV-A and SSC-A) (panel i), and dead cells were excluded by staining and gating for negative cells with FVS450 (panel ii). Immune cells and epithelial cells were excluded by gating the double negative population (CD45 (APC-$Cy7)^-$/EpCAM $(PE)^-$) (panel iii). Finally, endothelial cells were excluded by gating for CD31 (APC)-negative cells (panel iv). (**g**) Representative FACS gating strategies for sorting $EpCAM^-$ $CD45^-$ $CD31^-$ $CD34^+$ murine stromal cells. Cells were gated with forward and sideward scatter (FSV-A and SSC-A) (panel i), and dead cells were excluded by staining and gating for negative cells with FVS450 (panel ii). Immune cells and endothelial cells were excluded by gating the double negative population (CD45 (APC-$Cy7)^-$/CD31 $(APC)^-$) (panel iii). Finally, stromal cells were sorted by gating for the EpCAM $(PE)^-$/CD34 $(FITC)^+$ population (panel iv). (**h**) Heat map of scRNAseq data with genes differentially expressed in Fibroblast (Fib) 1, 2, and 3 of *in vivo* colon stroma.

Scale bars: 100 μm. Data are presented as mean ± SEM. Statistical analyses were performed using Student's *t*-test (two-tailed) for **a**, **d**, and **e**. scRNA-Seq data of *in vivo* mouse colon stromal cells are from published datasets GSE114374 (three biological replicates) and GSE172261 (six biological replicates). Images are representative of at least three biological replicates. Source data are provided as a Source Data file.

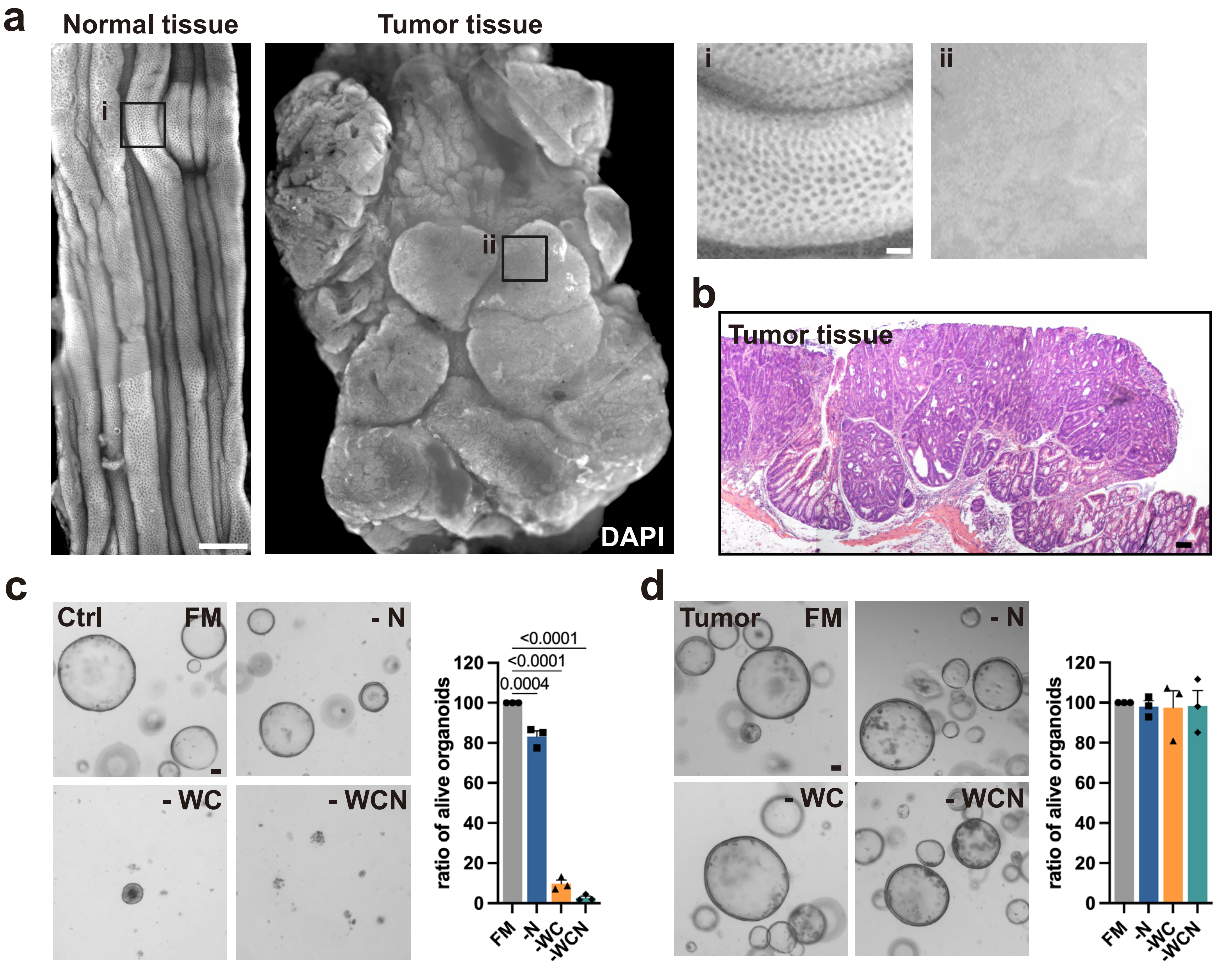

a
Normal tissue
Tumor tissue
i
ii
DAPI
b
Tumor tissue
c
Ctrl
FM
- N
- WC
- WCN
ratio of alive organoids
0
20
40
60
80
100
120
<0.0001
<0.0001
0.0004
FM
-N
-WC
-WCN
d
Tumor
FM
- N
- WC
- WCN
ratio of alive organoids
0
20
40
60
80
100
120
FM
-N
-WC
-WCN

Supplementary Figure 8. Establishment of AOM/DSS colitis-associated tumor model. (**a**) Whole-mount DAPI staining of normal colon tissue and AOM/DSS tumor tissue. Scale bars: 1 mm (high magnification: 100 μm). (**b**) Hematoxylin and eosin (H&E) staining of AOM/DSS tumor tissue. Scale bar: 100 μm. (**c** - **d**) Bright-field images and quantification of normal colon organoids (Ctrl) and colon tumor organoids cultured in full medium (FM), noggin-free (-N), sWnt-CHIR99021-free (-WC), or sWnt-CHIR99021-noggin-free (-WCN) media (*n* = 3 biological replicates per group). Scale bar: 100 μm. Data are presented as mean ± SEM. Statistical analyses were performed using Student's *t*-test (two-tailed) for **c** and **d**. Images are representative of at least three biological replicates. Source data are provided as a Source Data file.
