# Supplementary material for: Establishment of gastrointestinal assembloids to study the interplay between epithelial crypts and their mesenchymal niche
Source: Nat Commun. 2023 May 25;14:3025. doi: 10.1038/s41467-023-38780-3 (PMC10212920; doi:10.1038/s41467-023-38780-3)
Supplement: Supplementary file 2 — Reporting Summary [file 41467_2023_38780_MOESM2_ESM.pdf]

nature portfolio

Corresponding author(s): Michael Sigal

Last updated by author(s): May 4, 2023

Reporting Summary

Nature Portfolio wishes to improve the reproducibility of the work that we publish. This form provides structure for consistency and transparency in reporting. For further information on Nature Portfolio policies, see our Editorial Policies and the Editorial Policy Checklist.

Statistics

For all statistical analyses, confirm that the following items are present in the figure legend, table legend, main text, or Methods section.

n/a	Confirmed	
☐	☒	The exact sample size (*n*) for each experimental group/condition, given as a discrete number and unit of measurement
☐	☒	A statement on whether measurements were taken from distinct samples or whether the same sample was measured repeatedly
☐	☒	The statistical test(s) used AND whether they are one- or two-sided<br>*Only common tests should be described solely by name; describe more complex techniques in the Methods section.*
☒	☐	A description of all covariates tested
☐	☒	A description of any assumptions or corrections, such as tests of normality and adjustment for multiple comparisons
☐	☒	A full description of the statistical parameters including central tendency (e.g. means) or other basic estimates (e.g. regression coefficient) AND variation (e.g. standard deviation) or associated estimates of uncertainty (e.g. confidence intervals)
☐	☒	For null hypothesis testing, the test statistic (e.g. *F*, *t*, *r*) with confidence intervals, effect sizes, degrees of freedom and *P* value noted<br>*Give P values as exact values whenever suitable.*
☒	☐	For Bayesian analysis, information on the choice of priors and Markov chain Monte Carlo settings
☒	☐	For hierarchical and complex designs, identification of the appropriate level for tests and full reporting of outcomes
☒	☐	Estimates of effect sizes (e.g. Cohen's *d*, Pearson's *r*), indicating how they were calculated

Our web collection on statistics for biologists contains articles on many of the points above.

Software and code

Policy information about availability of computer code

Data collection	FCS Express 7 (De Novo)<br>Leica Application Suite X 3.5.6.21594 (LAS X, Leica)<br>QuantStudio 3 Real-Time PCR Software v1.7.1 (Thermo Fisher)<br>ZEN 3.4<br>Adobe Illustrator 26.3.1
Data analysis	ImageJ 2.0.0<br>GraphPad Prism 8<br>RStudio 2021.09.1<br>R 4.2.2<br>R packages (Seurat v3, GSVA 1.48.0, pheatmap 1.0.12, Harmony 0.1.153)<br><br>All computational codes can be accessed under https://github.com/Sigal-Lab/Lin_Colon_Assembloids.

For manuscripts utilizing custom algorithms or software that are central to the research but not yet described in published literature, software must be made available to editors and reviewers. We strongly encourage code deposition in a community repository (e.g. GitHub). See the Nature Portfolio guidelines for submitting code & software for further information.

Data

Policy information about availability of data

All manuscripts must include a data availability statement. This statement should provide the following information, where applicable:

- Accession codes, unique identifiers, or web links for publicly available datasets
- A description of any restrictions on data availability
- For clinical datasets or third party data, please ensure that the statement adheres to our policy

All scRNAseq data generated in this study have been deposited in the National Centre for Biotechnology, Gene Expression Information Omnibus (GEO) under accession code GSE231716 [https://www.ncbi.nlm.nih.gov/geo/query/acc.cgi?acc=GSE231716]. The previously published scRNAseq data are available in the GEO under accession code GSE114374 [https://www.ncbi.nlm.nih.gov/geo/query/acc.cgi?acc= GSE114374] and GSE172261 [https://www.ncbi.nlm.nih.gov/geo/query/acc.cgi?acc= GSE172261]. Source data are provided with this paper.

Research involving human participants, their data, or biological material

Policy information about studies with human participants or human data. See also policy information about sex, gender (identity/presentation), and sexual orientation and race, ethnicity and racism.

Reporting on sex and gender	NA
Reporting on race, ethnicity, or other socially relevant groupings	NA
Population characteristics	NA
Recruitment	NA
Ethics oversight	NA

Note that full information on the approval of the study protocol must also be provided in the manuscript.

Field-specific reporting

Please select the one below that is the best fit for your research. If you are not sure, read the appropriate sections before making your selection.

☒ Life sciences ☐ Behavioural & social sciences ☐ Ecological, evolutionary & environmental sciences

For a reference copy of the document with all sections, see nature.com/documents/nr-reporting-summary-flat.pdf

Life sciences study design

All studies must disclose on these points even when the disclosure is negative.

Sample size	No statistical methods were used to predetermine sample size. All experiments were performed with n = 3 biological replicates except where stated otherwise. Sample sizes were chosen based on available resources and community standards.
Data exclusions	No data were excluded for the analysis.
Replication	All attempts at replication were successful. All graphs represent data with at least two biological replicates. All images represent findings reproduced at least two times in the laboratory.
Randomization	The mice and assembloids/organoids/stromal cells were randomized for respective treatments.
Blinding	Group allocation and analysis were done in a blinded fashion.

Reporting for specific materials, systems and methods

We require information from authors about some types of materials, experimental systems and methods used in many studies. Here, indicate whether each material, system or method listed is relevant to your study. If you are not sure if a list item applies to your research, read the appropriate section before selecting a response.

Materials & experimental systems

n/a	Involved in the study
☐	☒ Antibodies
☒	☐ Eukaryotic cell lines
☒	☐ Palaeontology and archaeology
☐	☒ Animals and other organisms
☒	☐ Clinical data
☒	☐ Dual use research of concern
☒	☐ Plants

Methods

n/a	Involved in the study
☒	☐ ChIP-seq
☐	☒ Flow cytometry
☒	☐ MRI-based neuroimaging

Antibodies

Antibodies used

Immunofluorescence staining:
Rabbit anti-KI67 (Cell Signaling Technology, 9129S, clone D3B5, 1:100), rabbit anti-keratin 20 (Cell Signaling Technology, 13063S, clone D9Z1Z, 1:100), rabbit anti-MUC2 C3 (a gift from Prof. Gunnar C. Hansson, 1:1000), rabbit anti-synaptophysin (Abcam, ab178412, clone EPR1097-2, 1:100), rabbit anti-active YAP1 (Abcam, ab205270, clone EPR19812, 1:200), mouse anti-E-cadherin (BD, 610181, clone 36, 1:200), goat anti-αSMA (Abcam, ab5694, 1:100); rabbit anti-vimentin (Cell Signaling Technology, 5741S, clone D21H3, 1:100); rabbit anti-CD31 (Cell Signaling Technology, 77699S, clone D8V9E, 1:100); rabbit anti-β3-tubulin (Cell Signaling Technology, 5568S, clone D71G9, 1:100); Alexa Fluor 647-conjugated phalloidin (Life Technologies, A22287, 1:100); mouse anti-MUC5AC (Invitrogen, 12178, clone 45M1, 1:100); Alexa Fluor 647-conjugated lectin GSII (Thermo Scientific, L32451, 1:100); rabbit anti-cleaved caspase 3 (Cell Signaling Technology, 9661S, clone Asp175, 1:100). AlexaFluor 488 donkey anti-mouse IgG (Jackson Immunoresearch, 715-546-150, 1:250); Cy3 donkey anti-rabbit IgG (Jackson Immunoresearch, 711-166-152, 1:250); AlexaFluor 647 donkey anti-goat IgG (Jackson Immunoresearch, 705-605-003, 1:250).

Flow Cytometry:
PE-conjugated rat anti-CD326 (EpCAM) (Miltenyi Biotec, 130-117-779, clone caa7-9G8, 1:200), APC-Cy7-conjugated rat anti-CD45 (BD, 557659, clone 30-F11, 1:200), APC-conjugated rat anti-CD31 (BD, 551262, clone MEC 13.3, 1:200), and FITC-conjugated rat anti-CD34 (BD, 560238, clone RAM34, 1:100)

Validation

All antibodies used in the study were commercially available and validated by the manufacturer for respective applications, except for rabbit anti-MUC2 C3 which was generated and validated by Ingemar Carlstedt lab (https://link.springer.com/article/10.1007/BF00702348) and Gunnar C. Hansson lab (https://www.pnas.org/doi/10.1073/pnas.0803124105). rabbit anti-MUC2 was used to stain murine goblet cells. Further validation data can be found under above links.

Validation information of commercial antibodies used in the study:
Rabbit anti-KI67 was used to stain murine proliferative cells. We detected specific staining with the expected pattern. Further validation data can be found under https://www.cellsignal.com/products/primary-antibodies/ki-67-d3b5-rabbit- mab/9129?_=1553612454453&Ntt=D3B5&tahead=true.
Rabbit anti-keratin 20 was used to stain murine colonocytes. We detected specific staining with the expected pattern. Further validation data can be found under https://www.cellsignal.com/products/primary-antibodies/keratin-20-d9z1z-xp-rabbit-mab/13063.
Rabbit anti-synaptophysin was used to stain murine enteroendocrine cells. We detected specific staining with the expected pattern. Further validation data can be found under https://www.abcam.com/products/primary-antibodies/synaptophysin-antibody-epr1097-2-ab178412.html.
Rabbit anti-active YAP1 was used to stain active YAP1 in murine colonic cells. We detected specific staining with the expected pattern. Further validation data can be found under https://www.abcam.com/products/primary-antibodies/active-yap1-antibody-epr19812-ab205270.html.
Mouse anti-E-cadherin was used to stain murine epithelial cells. We detected specific staining with the expected pattern. Further validation data can be found under http:// www.bdbiosciences.com/us/applications/research/stem-cell-research/cancer-research/human/purified-mouse-anti-e- cadherin-36e-cadherin/p/610181.
Goat anti-αSMA was used to stain murine smooth muscle cells and myofibroblasts. We detected specific staining with the expected pattern. Further validation data can be found under https://www.abcam.com/products/primary-antibodies/alpha-smooth-muscle-actin-antibody-ab5694.html.
Rabbit anti-vimentin was used to stain murine fibroblasts. We detected specific staining with the expected pattern. Further validation data can be found under https://www.cellsignal.de/products/primary-antibodies/vimentin-d21h3-xp-rabbit-mab/5741.
Rabbit anti-CD31 was used to stain murine endothelial cells. We detected specific staining with the expected pattern. Further validation data can be found under https://www.cellsignal.com/products/primary-antibodies/cd31-pecam-1-d8v9e-xp-rabbit-mab/77699.
Rabbit anti-β3-tubulin was used to stain murine neuronal cells. We detected specific staining with the expected pattern. Further validation data can be found under https://www.cellsignal.com/products/primary-antibodies/b3-tubulin-d71g9-xp-rabbit-mab/5568?site-search-type=Products&N=4294956287&Ntt=5568s&fromPage=plp&_requestid=1375264.
Alexa Fluor 647-conjugated phalloidin was used to stain F-actin in murine cells. We detected specific staining with the expected pattern. Further validation data can be found under https://www.thermofisher.com/order/catalog/product/A22287.
Mouse anti-MUC5AC was used to stain murine gastric mucous pit cells. We detected specific staining with the expected pattern. Further validation data can be found under https://www.thermofisher.com/antibody/product/MUC5AC-Antibody-clone-45M1-Monoclonal/MA5-12178)
Alexa Fluor 647-conjugated lectin GSII was used to stain murine gastric base secretory cells. We detected specific staining with the expected pattern. Further validation data can be found under https://www.thermofisher.com/order/catalog/product/L32451.
Rabbit anti-cleaved caspase 3 was used to stain murine apoptotic cells. We detected specific staining with the expected pattern. Further validation data can be found under https://www.cellsignal.com/products/primary-antibodies/cleaved-caspase-3-asp175-antibody/9661?site-search-type=Products&N=4294956287&Ntt=9661s&fromPage=plp&_requestid=1375906.

PE-conjugated rat anti-CD326 was used to stain murine epithelial cells. We detected specific staining with the expected pattern. Further validation data can be found under https://www.miltenyibiotec.com/DE-en/products/cd326-epcam-antibody-anti-mouse-caa7-9g8.html#conjugate=pe:size=30-ug-in-200-ul.
APC-Cy7-conjugated rat anti-CD45 was used to stain murine hematopoietic cells. We detected specific staining with the expected pattern. Further validation data can be found under https://www.bdbiosciences.com/en-de/products/reagents/flow-cytometry-reagents/research-reagents/single-color-antibodies-ruo/apc-cy-7-rat-anti-mouse-cd45.557659.
APC-conjugated rat anti-CD31 was used to stain murine endothelial cells. We detected specific staining with the expected pattern. Further validation data can be found under https://www.bdbiosciences.com/en-de/products/reagents/flow-cytometry-reagents/research-reagents/single-color-antibodies-ruo/apc-rat-anti-mouse-cd31.551262.
FITC-conjugated rat anti-CD34 was used to stain CD34 in murine cells. We detected specific staining with the expected pattern. Further validation data can be found under https://www.bdbiosciences.com/en-de/products/reagents/flow-cytometry-reagents/research-reagents/single-color-antibodies-ruo/fitc-rat-anti-mouse-cd34.560238.

Animals and other research organisms

Policy information about studies involving animals; ARRIVE guidelines recommended for reporting animal research, and Sex and Gender in Research

Laboratory animals	C57BL/6 mice were obtained from Charles River Laboratory; tg Act-DsRed, tg Act-CFP, and Rosa26-tdTomato reporter mice were described previously. For lineage tracing of cells derived from AXIN2-expressing cells, Axin2CreERT2/Rosa26-tdTomato mice were generated by breeding Axin2CreErt2 to Rosa26-tdTomato mice. Bmpr1afl/fl mice were obtained from the laboratory of Yuji Mishina. To generate conditional KO mice with depletion of Bmpr1a in AXIN2+ cells, we bred Bmpr1afl/fl mice to Axin2CreERT2 mice. All animals were maintained in autoclaved micro-isolator cages and provided with sterile drinking water and chow ad libitum. The mice were bred at the animal care facility on a 12-h light/12-h dark cycle in a controlled temperature (22.5 ± 2.5 °C) and humidity (50 ± 5%) environment. Male 6 to 12-week-old mice were used for this study.
Wild animals	No wild animals were used in the study.
Reporting on sex	Only male animals were used in the study.
Field-collected samples	No filed-collected samples were used in the study.
Ethics oversight	All procedures involving animals were approved by the institutional, local, and national legal authorities (LaGeSo Berlin, T-CH 0032/20) at the Charité Universitätsmedizin Berlin.

Note that full information on the approval of the study protocol must also be provided in the manuscript.

Flow Cytometry

Plots

Confirm that:

☒ The axis labels state the marker and fluorochrome used (e.g. CD4-FITC).

☒ The axis scales are clearly visible. Include numbers along axes only for bottom left plot of group (a 'group' is an analysis of identical markers).

☒ All plots are contour plots with outliers or pseudocolor plots.

☒ A numerical value for number of cells or percentage (with statistics) is provided.

Methodology

Sample preparation	Preparation of stromal cells derived from colon tissue: The mouse colon was dissected, opened longitudinally, and cut into 1 cm long pieces. Tissue was washed three times in 1x phosphate-buffered saline (PBS) (Gibco), followed by incubation for 20 min in 10 mM EDTA (Invitrogen)/PBS supplemented with 0.5 mM Dithiothreitol (DTT) (Sigma) at 37 °C. The tissue fragments were washed four times with ice-cold PBS and shaken after every wash to get rid of epithelium. When clean supernatant was obtained, the tissue fragments were cut into tiny pieces and incubated in calcium and magnesium-free HBSS (Gibco) containing Liberase TL (1 unit/ml; Roche) and DNase I (1 unit/ml; Invitrogen) at 37 °C for 1 hour, with pipetting every 10 min. Every 20 min, the digested fraction was collected and put into an ice-cold stromal cell medium containing Advanced DMEM/F12 (Gibco), 10% fetal bovine serum (FBS, Gibco), 100 U/ml penicillin/streptomycin (Gibco), and 10 µM Y-27632 (Hoelzel). The collected fraction was filtered through a 70-µm cell strainer (Falcon) and centrifuged at 400 g for 5 min at 4 °C.<br><br>Preparation of primary colonic stromal cells derived from cell culture: Stromal cells were treated with TrypLE (Gibco) and incubated for 10-15 min at 37 °C. The dissociated cells were washed with Advanced DMEM/F-12 (Gibco) and centrifuged at 400g for 5 min at 4 °C.
Instrument	FACS Aria III (BD)<br>Cytek Aurora 3 laser spectral cytometer (CyTek Biosciences)
Software	FCS Express 7 (De Novo)

Cell population abundance

The purity of cells was verified by re-sorting of sorted cells during the establishment of the sorting protocol for the experiments presented here and was confirmed to be more than 95%.

Gating strategy

Gating strategies for sorting EpCAM- CD45- CD31- CD34+ murine stromal cells: Cells were gated with forward and sideward scatter (FSV-A and SSC-A), doublets were excluded by gating with SSC-A vs. SSC-H and FSC-A vs. FSC-H, respectively, and dead cells were excluded by staining and gating for negative cells with FVS450. Immune cells and endothelial cells were excluded by gating the double negative population (CD45 (APC-Cy7)-/CD31 (APC)-). Finally, stromal cells were sorted by gating for the EpCAM (PE)-/CD34 (FITC)+ population (panel 4).

Gating strategies for analyses of alive (FVS450-) EpCAM (PE)- CD45 (APC-Cy7)- CD31 (APC)- primary murine colonic stromal subsets. Cells were gated with forward and sideward scatter (FSV-A and SSC-A), doublets were excluded by gating with SSC-A vs. SSC-H and FSC-A vs. FSC-H, respectively, and dead cells were excluded by staining and gating for negative cells with FVS450. Immune cells and epithelial cells were excluded by gating the double negative population (CD45 (APC-Cy7)-/EpCAM (PE)-). Finally, endothelial cells were excluded by gating for CD31 (APC)-negative cells (panel 4).

☒ Tick this box to confirm that a figure exemplifying the gating strategy is provided in the Supplementary Information.
